# Active substances of myxobacteria against plant diseases and their action mechanisms

**DOI:** 10.3389/fmicb.2023.1294854

**Published:** 2024-01-08

**Authors:** Lele Zhang, Liangliang Bao, Songyuan Li, Yang Liu, Huirong Liu

**Affiliations:** ^1^College of Life Sciences, Inner Mongolia Agricultural University, Hohhot, Inner Mongolia, China; ^2^College of Science, Inner Mongolia Agricultural University, Hohhot, Inner Mongolia, China

**Keywords:** myxobacteria, plant diseases, biological control, carbohydrate-active enzymes, small molecule compounds

## Abstract

Myxobacteria have a complex life cycle and unique social behavior. They can prey on plant pathogenic fungi, bacteria, and oomycetes in the soil by producing some enzymes and small molecule compounds. The enzymes mainly include β-1,6-glucanase, β-1,3-glucanase, chitinase, protease, peptidase, and formaldehyde dismutase. β-1,6-glucanase, β-1,3-glucanase, and chitinase can degrade the glycosidic bonds in the cell wall of plant pathogen, causing some holes to form on the cell walls of the plant pathogen. Proteases and peptidases can break plant pathogenic cells into many small fragments and facilitate extracellular digestion of proteins during myxobacterial predation. Formaldehyde dismutase converts formaldehyde to formate and methanol, it can help myxobactria protect themselves in the process of predation. Small molecule substances produced by myxobacteria include isooctanol, di-isobutyl phthalate, myxovirescin, cystobactamid derivatives, hyalodione, argyrin derivatives, Methyl (2R)-2-azido-3-hydroxyl-2-methylpropanoate and N-(3-Amino-2-hydroxypropyl)-N-meth-ylsulfuric diamide, etc. Isooctanol destroyed the cell wall and cell membrane of plant pathogen, causing intracellular reactive oxygen species (ROS) to accumulate, leading to apoptosis and cell death. Di-isobutyl phthalate had biofilm inhibitory activity against bacteria. Myxovirescin could inhibit the incorporation of diamibopimelic acid and uridine diphosphate-Nacetylglucosamine intobacterial cell wall and interfered with the polymerizaton of the lipid-disacchar-pentapeptide. Cystobactamid derivatives exerted their natural antibacterial properties by inhibition of bacterial gyrases. Hyalodione had broad antibacterial and antifungal activity. Argyrin derivatives inhibited protein synthesis by interfering with the binding of elongation factor G (EF-G) to ribosomes. Methyl (2R)-2-azido-3-hydroxyl-2-methylpropanoate and N-(3-Amino-2-hydroxypropyl)-N-meth-ylsulfuric diamide reduced the content of soluble proteins and the activity of protective enzymes (PPO, POD, PAL, and SOD) in plant pathogen, increased oxidative damage and cell membrane permeability. Myxobacteria, as a new natural compound resource bank, can control plant pathogenic fungi, oomycetes and bacteria by producing some enzymes and small molecule compounds, so it has great potential in plant disease control.

## 1 Introduction

Myxobacteria are microorganisms of the phylum Myxococcota (Waite et al., 2020; Oren and Garrity, 2021), which are well known for their complex life cycles and unique social behaviors. Myxobacteria have a wide range of habitats, including soil rich in organic matter, rotting wood, animal dung and marine environment (Saggu et al., 2023). They can survive in high-salinity environments (Gemperlein et al., 2018). Some halophilic myxobacteria i.e., *Haliangium spp*. (Ryosuke et al., 2002), *Plesiocystis pacifica* (Iizuka et al., 2003a) and *Enhygromyxa salina* (Iizuka et al., 2003b) had been isolated from marine environment. Research over the past few decades has proven that myxobacteria have become a resource library of new natural compounds, ranking second only to *Actinomycetes* and *Bacillus* among prokaryotes (Arguelles-Arias et al., 2009; Weissman and Müller, 2010). Metabolites produced by myxobacteria often have structures that other microbial metabolites do not have, and40% of myxobacterial metabolites have novel chemical structures. For example, in contrast to Actinomycetes derivatives, most small molecules of myxobacteria are not glycosylated (Rix et al., 2002). It is currently unclear why myxobacteria produce large amounts of metabolites, but researchers generally believe that metabolites play an important role in regulating cell-to-cell interactions within a population (Davies et al., 2006) and in prey hunting (Xiao et al., 2011).

Myxobacteria can prey on plant pathogen and destroy pathogen’s cell morphology and structure. When myxobacteria prey on pathogen, they can kill microorganisms and lyse cells by producing metabolites such as antibiotics, cell wall degrading enzymes, lipases, nucleases, polysaccharases, and proteases, thereby clearing the pathogens. The destroyed pathogenic cells are surrounded by many filamentous substances. The cell structure become loose and irregular, and the cell contents overflow, and eventually the pathogen lyse and die.

Therefore, myxobacteria can serve as biological control agents (BCAs) of plant diseases (Ye et al., 2020b). The BCAs in agricultural planting can reduce the use of pesticides, reduce the adverse effects caused by excessive use of chemicals and achieve the purpose of controlling soil-borne plant diseases. The BCAs are very effective in preventing and managing plant diseases and achieving ecological and economic benefits such as increasing agricultural output and reducing environmental pollution. Research on myxobacteria can provide new potential ways for biological control of plant diseases. This paper reviews the research progress on the active substances of myxobacteria against plant diseases and their action mechanisms.

## 2 Enzymes

**Figure 1:**
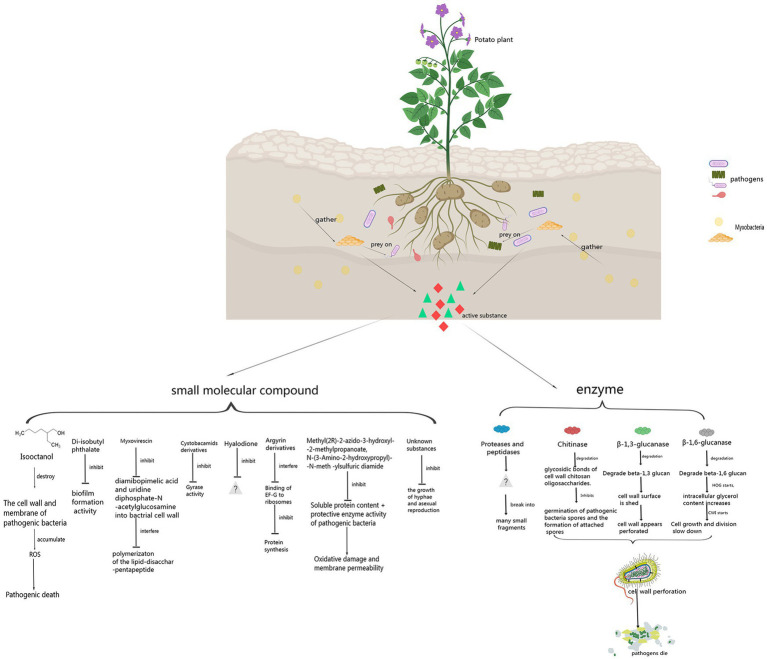
Control principle of myxobacteria against pathogens.

Myxobacteria produce some enzymes playing important roles in preying on pathogens. These enzymes include carbohydrate-active enzymes (CAZymes), peptidases, lipases, etc. CAZymes include glycosyltransferases (GTs), glycoside hydrolases (GHs), carbohydrate esterases (CEs), auxiliary activities (AAs), carbohydrate-binding modules (CBMs), and polysaccharide lyases (PLs). CAZymes can modify the glycosidic bonds of carbohydrates and are important basic functional units in carbohydrate metabolism pathways (Dong et al., 2023). GHs hydrolyze glycosidic bonds and play an important role in the hydrolysis and synthesis of sugars and glycoconjugates in organisms (Kaushal and Singh, 2020). The enzymes produced by myxobacteria to control plant pathogens are shown in Figure 1 and Table 1.

**Table 1:** Control principle of myxobacteria against pathogens.

Antagonistic substances	Antagonistic principle	References
**Enzymes**
β-1,6-Glucanase	Degrades β-1,6-glucan. When HOG is activated, the intracellular glycerol content increases and the osmotic pressure increases; when CWI is activated, cell growth and division slow down.	Zhou et al. (2019) and Ye et al. (2020a)
β-1,3-Glucanase	Hydrolyzesβ-1,3-glucan. The cell wall surface falls off and a perforated structure appears in the cell wall.	Zhou et al. (2019) and Zhang et al. (2023)
Chitinase	Degrades the glycosidic bonds of cell wall chitosan oligosaccharides. Inhibits the germination of pathogenic bacteria spores and the formation of attached spores.	Li et al. (2019a)
Proteases and peptidases	The morphology of R. solanacearum changed significantly, and the cells were broken into many small fragments	Dong et al. (2023)
Formaldehyde dismutase	Decompose the formaldehyde produced by pathogenic bacteria to prey on them.	Willsey et al. (2016) and Sutton et al. (2019)
**Small molecule compounds**
Isooctanol	Destroy the cell wall and cell membrane of pathogenic bacteria, accumulate ROS, and cause cell death.	Ye et al. (2020a)
Di-isobutyl phthalate	Exhibits biofilm formation inhibitory activity against pathogenic bacteria.	Sharma et al. (2021)
Myxovirescin	Inhibits the incorporation of diamibopimelic acid and uridine diphosphate-N-acetylglucosamine into bactrial cell wall, and interferes with the polymerizaton of the lipid-disacchar-pentapeptide	Rosenberg et al. (1982) and Paitan et al. (1999)
Cystobacamids derivatives	Inhibits bacterial gyrase to exert antibacterial activity	Kim et al. (2019) and Solga et al. (2024)
Hyalodione	Strongly inhibits the activity of pathogenic bacteria.	Okanya et al. (2012)
Argyrin derivatives	Participate in the non-ribosomal peptide synthase pathway and inhibit protein synthesis by interfering with the binding of elongation factor G (EF-G) to ribosomes	Pogorevc et al. (2019) and Wieland et al. (2022)
Methyl(2R)-2-azido-3-hydroxyl-2-methylpropanoate, N-(3-Amino-2-hydroxypropyl)-N-meth-ylsulfuric diamide	Reduce the content of soluble proteins and activity of protective enzymes in pathogenic bacteria, and increase oxidative damage and cell membrane permeability.	Wu (2018)
Some unknown substances	Inhibits the growth of hyphae and asexual reproduction of Phytophthora infestans	Ren et al. (2016), Ding (2017), Wu (2018) and Zhao (2018)

### 2.1 β-1,6-Glucanase

β-1,6-glucan is a component of the fungal cell wall smaller than chitin and β-1,3-glucan. It can cross-link cell wall proteins to the chitin layer and β-1,3-glucan layer. Inhibiting the synthesis of β-1,6-glucan is conducive to the effective disintegration and further degradation of pathogen cell wall during the process of myxobacteria preying on plant pathogen. β-1,6-glucanase can hydrolyze the glycosidic bonds of β-1,6-glucan, thereby destroying the entire cell wall structure of fungi. β-1,6-glucanase GluM from the strain EGB of *Coralococcus* sp. is a novel family of outer membrane β-barrel proteins that can inhibit fungal embryonic tube development (Li et al., 2019b). β-1,6-glucanase GluM is essential in the initial sensing and efficient decomposition of fungi.

Electron microscopy observation of the hyphae of *Magnaporthe oryzae* treated with β-1,6-glucanase GluM showed that the hyphae were stretched and partially broken, and the hyphal cell wall changed from a dense structure to a loose structure. The spore folds of the treated *M. oryzae* were irregular. The density of spore decreased, and the morphology of spore showed a deformed state. The morphological and structural changes of *M. oryzae* were speculated to be due to the hydrolysis of the cell wall by β-1,6-glucanase GluM, resulting in incomplete cell structure and outflow of contents, ultimately leading to morphological changes. Therefore, β-1,6-glucanase GluM inhibited the infection of *M. oryzae* in rice by digesting the pathogen’s cell wall (Zhou et al., 2019).

After treated with GluM, the hyphae and spores of *Fusarium oxysporum f*. sp. *cucumerinum* (FOC) shrank obviously. The cell wall of FOC appeared to be perforated and damaged, the cell wall structure was loose, and large vacuoles formed in the cells. The High Osmolarity Glycerol (HOG) in FOC cells was activated. The phosphorylation level of Hog1-likemitogenactivated proteinkinase (MAPK) was significantly increased and the glycerol content increased 2.6 times. The osmotic pressure in FOC cells increased, which accelerated cell lysis. When the strain EGB of *Corallococcus* sp. were inoculated with the potted cucumbers, the strain could adapt well to the soil environment and effectively reduced the abundance of soil-borne *F. oxysporum* and the occurrence of cucumber wilt disease (Ye et al., 2020b).

In the *GluM* transgenic experiment, the β-1,6-glucanase gene was transferred into japonica rice variety ZH11 to obtain transgenic japonica rice with overexpression of *GluM*. In the fungal disease resistance experiment, the rice blast area of *GluM* transgenic rice was reduced by 82.7%. The sheath blight disease was reduced by 35.76%−43.67% and the incidence of rice smut disease was reduced by 65.79%. The results showed that transgenic rice containing GluM protein could degrade fungal cell walls through specific hydrolysis and enhanced resistance to fungal diseases (Shen et al., 2023).

The β-1,6-glucanase produced by myxobacteria inhibits not only the growth of pathogenic fungus, but also the growth of oomycetes. The fermentation products of *C. coralliformis* strain CMC0606 had a strong inhibitory effect on *Phytophthora capsici*, and the diameter of inhibition zone was 16mm (Bader et al., 2022). The strain EGB had a strong inhibitory effect on the growth of *P. capsica*. The mycelium of *P. capsica* collapsed and the growth of the pathogen was obviously inhibited. The results showed that β-1,6-glucanase in the fermentation supernation of strain EGB was effective in inhibiting oomycetes (Zhang et al., 2023).

### 2.2 β-1,3-Glucanase

β-1,3-glucan is a component of the fungal cell wall. Extensive hydrolysis of fungal cell wall polymer chains by β-1,3-glucanase can reduce the mechanical strength of the cell wall, leading to the final lysis of the fungal cell. β-1,3-glucanase IamC from strain EGB can cleave β-1,3- or β-1,6-glucan substrates by exo-hydrolysis. Cu^2+^, Co^2+^, Mg^2+^, and Cr^3+^ inhibit the activity of IamC, while Mn^2+^ is an effective activator of IamC, indicating that IamC is a metal ion-dependent hydrolase. After exposure of *M. oryzae* to IamC, the germ tube and appressorium formation rates were significantly reduced from 94 and 97% to 59 and 51%. The hyphae of *M. oryzae* was enlarged and deformed, and more granular contents appeared inside the hyphae. There was a large accumulation of reactive oxygen species (ROS) in the spores and hyphae of *M. oryzae* and the distribution of chitin in the cell wall of pathogen changed. The β-1,3-glucanase IamC derived from strain EGB acted on different β-glycosidic bonds in the cell wall of *M. oryzae* . β-glycosidic bonds of polysaccharides from different sites of cell wall of pathogen were hydrolyzed, ultimately leading to cell lysis of *M. oryzae* (Zhou et al., 2019).

*Archangium* strain AC19 showed strong predatory activity gainst *Phytophthora sojae* P6497 and protected soybeans from stem rot disease. Strain AC19 was observed to prey on *P. sojae*, and the hyphal cell wall of *P. sojae* P6497 showed perforation. The fermentation supernatant of strain AC19 significantly inhibited the growth and infection of *P. sojae*. The active substances that digested *P. sojae* were the CAZymes secreted by strain AC19. The cell wall-acting CAZymes in strain AC19 were specialized β-1,3-glucanases (AcGlu13.1,−13.2, and−13.3). These β-1,3-glucanases targeted β-1,3-glucan from the cell wall of *Phytophthora*. AcGlu13.1 caused cell wall surface shedding through its degradative activity. AcGlu13.2 and AcGlu13.3 could cause perforated structures in the cell wall (Zhang et al., 2023).

### 2.3 Chitinase

The fungal cell wall is mainly composed of chitin, β-1,3-glucan and β-1,6-glucan. The chitin accounts for 22%−40% of the fungal cell wall (Bowman and Free, 2006). Chitinase ofGHs family can hydrolyze chitin in fungal cell walls, so chitinase is regarded as an antifungal factor for biocontrol of fungal diseases (Shehata et al., 2018; Li et al., 2019a). Strain EGB can synthesize the endo-chitinase CcCti1 which belongs to the GHs family 18 (GH18) and has potential antifungal activity. CcCti1 can not only degrade chitosan oligosaccharide, but also hydrolyze chitin into N-acetylated chitohexaose (GlcNAc)6. CcCti1 had biological control activity against the plant pathogen *M. oryzae*, inhibiting the germination of conidia and the formation of appressoria of *M. oryzae* at a concentration of 0.08 mg/mL (Li et al., 2019a). Rice blast caused by *M. oryzae* is the main limiting factor in global rice production and is one of the most destructive diseases in cultivated rice in the world (Talbot, 2003). The transgenic plants with chitinase genes showed strong resistance to rice blast. The reason may be that the cell wall integrity of pathogen changed, leading to significant internal expansion pressure and lysis of fungal cells (Selitrennikoff, 2001). Therefore, transgenic plants with chitinase genes were more resistant to the *M. oryzae*.

### 2.4 Proteases and peptidases

Tomato bacterial wilt (TBW) caused by *Ralstonia solanacearum* is one of the most destructive soilborne diseases, and tomato production has suffered huge losses due to the epidemic of TBW (Mansfield et al., 2012). *M. xanthus* R31 had good biological control potential against TBW, and the biocontrol efficiency against TBW in pot experiments was as high as 81.9%. The MEROPS database of strain R31 genome had annotated 274 proteins, including 132 metalloproteases and 107 serine proteases. Three M36 metalloproteases were identified in the R31 genome that may contribute to the extracellular digestion of proteins during predatory behavior (Dong et al., 2023). Proteins of the M23 family were endopeptidases that cleaved bacterial cell wall peptidoglycan by degrading the peptide bonds of cross-linked peptides (Odintsov et al., 2004). Proteases and peptidases, such as the M36 metalloprotease MepA secreted by *M. xanthus* strain DK1622, may promote the predation of prey by degrading proteins of prey cells (Berleman et al., 2014).

### 2.5 Formaldehyde dismutase

When *Pseudomonas aeruginosa* was preyed on, it secreted toxic formaldehyde to resist predation. Fomaldehyde can be converted into formate and methanol by formaldehyde detoxifying enzymes, such as formaldehyde dismutase (Fdm), produced by *P. aeruginosa* to protect itself (Willsey et al., 2016). It was shown that myxobacteria could produce formaldehyde dismutase. Therefore, myxobacteria had the ability to prey on *P. aeruginosa* by converting toxic formaldehyde secreted by *P. aeruginosa* into non-toxic substances (Sutton et al., 2019). *Myxobacteria* may also be able to prey on similar plant pathogens by a similar way.

## 3 Small molecule compounds

The biological control activity of *myxobacteria* against pathogen depends on not only enzymes, but also some small molecule compounds. These compounds include isooctanol, di-isobutyl phthalate, myxovirescin, cystobactamid derivatives, hyalodione, argyrin derivatives, Methyl (2R)-2-azido-3-hydroxyl-2-methylpropanoate, and N-(3-Amino-2-hydroxypropyl)-N-meth-ylsulfuric diamide, etc. Some small molecule compounds produced by myxobacteria to control plant pathogens are shown in Figure 1 and Table 1.

### 3.1 Isooctanol

Strain EGB exhibited superior biological control activity against *F. oxysporum*. *F. oxysporum* is a ubiquitous soil-borne plant pathogen that can cause vascular wilt in a variety of crops (Pietro et al., 2003). A total of 32 volatile compounds produced by strain EGB were identified, and isooctanol had the highest antifungal activity. The mycelia of *F. oxysporum* treated with isooctanol showed severely shrinkage and collapse. The hyphae of *F. oxysporum* treated by isooctanol, the transcript levels of many genes related to the cell wall integrity (CWI) pathway and redox reactions were significantly increased by 15-to 40-fold. The transcription levels of chitin synthase (FOXG_12345, FOXG_10443 and FOXG_04179), chitinase (FOXG_19879 and FOXG_17332), endo-1,3 (4)-β-glucanase (FOXG_22849, FOXG_10637 and FOXG_03928) were upregulated after the mycelia of *F. oxysporum* were treated with isooctanol. The transcription levels of genes related to components corresponding to cell wall integrity (FOXG_09228), programmed cell death control protein (FOXG_03587) and cell division control protein (FOXG_00362) increased, slowing down the growth and division rate of cells, and activating cell apoptosis. Isooctanol destroyed the cell wall and cell membrane of *F. oxysporum*, causing intracellular reactive oxygen species (ROS) to accumulate, leading to apoptosis and cell death. A dose of only 3.75 μL/plate of isooctanol was sufficient to inhibit *F. oxysporum* (Ye et al., 2020a).

### 3.2 Diisobutyl phthalate

*M. fulvus* strain ST/P/71 had obvious antibacterial activity against *B. subtilis*, and the extract from the strain ST/P/71 mainly showed inhibition activity against *B. subtilis*. During seperating by Reverse Phase High Performance Liquid Chromatography (RP-HPLC), two pure compounds were eluted at RT 54.24 (Ra2) and RT 71.27 (Ra3). Ra2 was identified as di-isobutyl phthalate. This substance showed biofilm formation inhibitory activity against *B. subtilis*, with an MBIC50 of 2.703 μg/mL (Sharma et al., 2021). Di-isobutyl phthalate had biofilm formation inhibitory activity against *B. subtilis*, so it could have similar functions to plant pathogenic bacteria. Therefore, diisobutyl phthalate may have great potential in the prevention and control of plant pathogenic bacteria.

### 3.3 Myxovirescin

One of the 18 metabolites of *M. xanthus* strain DK1622 is polyketide *myxovirescin* (antibiotic TA), which had antibacterial activity. *Myxovirescin* played an important role in killing *Escherichia coli*, including lysis and subsequent predation (Xiao et al., 2011). The antibiotic TA was produced and named after *M. vanthits* strain TA(ATCC31046) (Rosenberg et al., 1982). The antibiotic TA could inhibit the incorporation of diamibopimelic acid and uridine diphosphate-N-acetylglucosamine into *E. coli* cell wall, and antibiotic TA interfered with the polymerizaton of the lipid-disacchar-pentapeptide (Rosenberg et al., 1982; Paitan et al., 1999). Myxobacteria encode a variety of substances to attack prey cells, with antibiotics serving as a front line weapon. Antibiotics can act as small molecule weapons to penetrate and kill or neutralize the metabolism of prey. The prevention and control principle of myxovirescin against bacterial pathogens has been relatively clear. This substance can be applied to research on prevention and control of plant pathogenic bacteria.

### 3.4 Cystobactamid derivatives

*Myxobacteria* produce specialized metabolites when preying, the production of specialized metabolites and lytic proteins of *myxobacteria* is related to their predation (Akbar and Stevens, 2021). Müller et al. found that the target compound inhibiting *P. aeruginosa* and other bacterial pathogens had a UV absorption spectrum similar to that of cystobactamides. Cystobacamids are aromatic oligoamides that exert their natural antibacterial properties by inhibition of bacterial gyrases (Solga et al., 2024). The improved orthogonally functionalized methoxyaspartate of cystobactamides could expand the synthesis of new cystobactamides. At present, four new types of cystobactamides 919-1 (1), 919-2 (2), 920-1 (3), 920-2 (4) and cystobactamides 861-2 (5) had been successfully synthesized and measured. Moeller et al. (2019) compared the antibacterial properties of this class of substanaces, the cyano derivative of cystobactamide 861-2(5) had antimicrobial activity against Gram-negative bacteria and its activity was higher than that of any natural cystobactamide tested so far.

Culture isolation of *C. coralline* strain M23 yielded coralmycin A (1), B (2) and another derivative, cystobactin 919-2. Coralmycin A had the strongest antibacterial activity against Gram-negative bacteria and had a wide antibacterial spectrum. Coralmycin A and B were cystobactamid derivatives (Kim et al., 2016). Seven new coralmycin derivatives and three known compounds were also isolated from the another culture of strain M23. The coralmycin derivatives are C (1), D (2), E (3), F (4), G (5), H (6), and I (7), the known compounds are cystobactamide 891-2(8), 905-2(9), and 507(10). The compounds had DNA gyrase inhibitory activity and antibacterial activity. The β-methoxyasparagine structure of coralmycin may affect prey ingestion (Kim et al., 2019). Research on the structure of cystobactamides can be used to develop new structural substances with more antibacterial activity. Therefore, this type of substances may also have great potential in the prevention and control of Gram-negative plant pathogenic bacteria.

### 3.5 Hyalodione

Hyalodione isolated from the extract of the *Hyalangium minutum* strain NOCB-2T had antibacterial activity against *P. aeruginosa*. Hyalodione is a novel S-methyl cyclohexadiene-dione, which belongs to the class qinone (Herrmann et al., 2017). Hyaladione had broad antibacterial and antifungal activity. The tested strains included *P. aeruginosa*, *Rhodotorula glutarum* and *Staphylococcus aureus* (Okanya et al., 2012). Research on how hyalodione control bacteria and fungi need to be continued. Hyalodione also has great potential in controlling plant pathogenic bacteria and fungi.

### 3.6 Argyrin derivatives

The culture medium of the strains of *Archangium gephyra* contained a group of cyclic peptides composed of naturally produced octapeptides, which exhibited strong antibiotic effects against *P. aeruginosa* (Nickeleit et al., 2008; Stauch et al., 2010; Wieland et al., 2022). Among them, argyrin participates in the non-ribosomal peptide synthetase pathway, combining with elongation factor G (EF-G) as its target (Pogorevc et al., 2019). Argyrins inhibit protein synthesis by interfering with EF-G binding to the ribosome (Wieland et al., 2022). Several derivatives of argyrin A could be obtained by modifying the methoxytry ptophan residue. The methoxy group of this residue was crucial for its antibacterial activity and its activity will be lost if the position is replaced by other substituents (Siebert et al., 2019). Argyrin has antibacterial activity against bacteria, so there is great practical value to study its antibacterial activity against plant pathogenic bacteria.

### 3.7 Methyl (2R)-2-azido-3-hydroxyl-2-methylpropanoate and N-(3-amino-2-hydroxypropyl)-N-meth-ylsulfuric diamide

The predation of bacteria and fungi by predatory *myxobacteria* has been mostly studied, but the predation of oomycetes has received little attention. *P. infestans* is the pathogen that causes potato late blight, which is one of the most serious diseases of potato (Rix et al., 2002). Wu collected *myxobacteria* isolated from soil samples in central Inner Mongolia. Eighty-three percent of the myxobacterial strains were resistant to *P. infestans*, among which the strains of *Myxococcus* and *Coralococcus* accounted for a higher proportion. The strains with the most significant antibacterial activity were *M. xanthus* B25-I-1, *M. fulvus* B25-I-3 and *M. stipitatus* X6-II-1. Strain B25-I-1 exhibited antagonistic activity against a variety of fungi and bacteria, and its active substances reduced the content of soluble proteins and the activity of protective enzymes (PPO, POD, PAL, and SOD) in *P. infestans*, increased oxidative damage and cell membrane permeability. It had a strong inhibitory effect on the hyphae, asexual reproduction and sexual reproduction of *P. infestans* (Wu, 2018). The active substance of strain B25-I-3 showed a strong inhibitory effect on the growth of *P. infestans*, inhibited the growth of mycelium and asexual reproduction, and reduced the infection ability of pathogens (Wu, 2018; Wu Z. et al., 2021; Wu Z. H. et al., 2021). After the fermentation products of B25-I-1 and B25-I-3 were separated, it was found that the components that had antagonistic effects on *P. infestans* contained Methyl(2R)-2-azido-3-hydroxyl-2-methylpropanoate and N-(3-Amino-2-hydroxypropyl)-N-meth-ylsulfuricdiamide.

### 3.8 Some unknown substances

*P. infestans* causes devastating diseases by invading the leaves, stems and tubers of potato plants (Berleman and Kirby, 2009). *M. xanthus* YR-7 isolated from soil samples in Bayannur area of Inner Mongolia had significant resistance to *P. infestans*. The growth inhibition rate of strain YR-7 against *P. infestans* hyphae was as high as 96.67%. The fermentation product of YR-7 was tested in isolated leaves. The experimental results proved that the active substance against *P. infestans* is a non-protein substance (Ren et al., 2016). About 72% of the myxobacteria isolated from soil samples in Ordos and Wuhai areas of Inner Mongolia had varying degrees of antagonistic effects on the growth of *P. infestans*. The ones with stronger ability to inhibit oomycetes were *C. exiguous* E10, *M. fallax* E11 and *C. coralloides* E12. The diameters of the inhibition zones were 26 mm, 24 mm and 24 mm (Ding, 2017; Wu, 2018). About 78.75% of the myxobacteria isolated from soil samples in Alxa area of Inner Mongolia had varying degrees of activity against *P. infestans*. The resistance of *Myxococcus* *fulvus* AL-24 and *Anqiococcu* AL-10 was outstanding. The fermentation products of strain AL-24 and strain AL-10 had good infection prevention activity and weak infection treatment activity on detached potato leaves (Zhao, 2018). Myxobacteria isolated from Indian soil samples, the extracts of *Coralococcus* *parvum* S104 and S145 showed broad-spectrum antibacterial activity. The water extract (WE) and DMSO extract (DE) of GNDU172 showed obvious activity against *B. subtilis* , and the DE of strain S213 and strain S223 showed similar activity. The DE of S223 has activity against *Pseudomonas syringae* and *B. cereus* (Kumar et al., 2017). However, all of these active substances are unknow and need to be seperated and identified in the future.

## 4 Discussion

Plant pathogen is a major threat to crops worldwide, not only reducing crop yields but also causing significant damage to crop quality. In order to control or avoid excessive economic losses caused by plant pathogens, synthetic agrochemicals are often used in agricultural production as the most common method to control plant pathogens and improve crop yield quality. However, excessive use can cause adverse effects on the environment and human health.

Myxobacteria feed on bacteria and fungi in the soil and obtain nutrients by preying on other microorganisms. The cell wall of plant pathogens protects cells from external invasion and is a key barrier between predatory myxobacteria and prey cells (Berleman and Kirby, 2009). In order to break through the cell wall barrier, Myxobacteria have evolved targeted preying methods. One method is to degrade the cell wall or increase the permeability of the cell membrane through antibacterial metabolites and cell wall degrading enzymes. The other one is a targeted, contactdependent killing mechanism through the Tad-like system and protein secretion system (Thiery et al., 2022). The Tad-like system mediates the contact-dependent killing of myxobacteria on prey cells (Seef et al., 2021).

The general method for myxobacteria to control plant pathogenic bacteria is the first method mentioned above. During the interaction between myxobacteria and prey cells, antibiotics, lytic enzymes, hydrolases, etc. involved in the process. This review summarizes the prevention and control principles of myxobacteria in plant pathogenic fungi, oomycetes, and bacterial disasters (Table 1). Myxobacteria secrete enzymes that can degrade cell wall components. Myxobacteria produce small molecule compounds that inhibit the normal growth and proliferation of pathogens. The enzymes secreted by myxobacteria are mainly chitinase, β-1,6-glucanase and β-1,3-glucanase, etc, which increase intracellular osmotic pressure by degrading the cell wall of prey cells, promoting cell lysis and death. Some small molecule compounds produced by myxobacteria affect the normal growth and reproduction of prey cells or changing their permeability, thereby inducing apoptosis of prey cells.

In the process of preventing and controlling plant diseases, it is possible to use myxobacteria to develop a certain BCAs that inhibits pathogens efficiently, which will be helpful to reduce the cost increase and environmental pollution caused by excessive use of pesticides, and maximize economic and environmental benefits.

In addition to preventing and controlling plant diseases in agriculture, myxobacteria can also be used to increase production, storage, and transportation of agricultural and sideline products. For example, myxobacteria-mediated paper impregnated with silver nanoparticles (AgNPs) is used in fruit packaging to extend the shelf life to 15 days (Bhople et al., 2016).

At present, there are only relevant reports on the prevention and control of plant diseases by myxobacteria on fungi, oomycetes and bacteria. There are no reports on the prevention and control of plant virus damage. The principle of prevention and treatment of animal viruses by myxobacteria is to inhibit the activity of RNA-dependent RNA polymerase complex, ion channel inhibitor, and signal pathway inhibition. Myxobacteria are also very likely to inhibit RNA synthesis or inhibit signaling pathways in the prevention and control of plant virus damage. Therefore, myxobacteria also have great research value in the prevention and control of plant virus damage.

## Author contributions

LZ: Conceptualization, Investigation, Software, Writing – original draft. LB: Methodology, Writing – review & editing. SL: Data curation, Formal analysis, Writing – original draft. YL: Resources, Writing – review & editing. HL: Project administration, Supervision, Writing – review & editing.
