# Corrigendum: Active substances of myxobacteria against plant diseases and their action mechanisms

**DOI:** 10.3389/fmicb.2024.1392109

**Published:** 2024-03-13

**Authors:** Lele Zhang, Liangliang Bao, Songyuan Li, Yang Liu, Huirong Liu

**Affiliations:** ^1^College of Life Sciences, Inner Mongolia Agricultural University, Hohhot, Inner Mongolia, China; ^2^College of Science, Inner Mongolia Agricultural University, Hohhot, Inner Mongolia, China

**Keywords:** myxobacteria, plant diseases, biological control, carbohydrate-active enzymes, small molecule compounds

In the published article, there was an error in the article title. Instead of “Substances derived from myxobacteria that prevent and control plant pathogenic diseases and their prevention and control principles,” it should be “Active substances of myxobacteria against plant diseases and their action mechanisms.”

1. In the published article, some references were not cited. The missing references are listed below. In the published article [Berleman, J.E., Allen, S., Danielewicz, M.A., Remis, J.P., Gorur, A., Cunha, J., et al. (2014). The lethal cargo of Myxococcus xanthus outer membrane vesicles. Frontiers in Microbiology 5. 10.3389/fmicb.2014.00474] was not cited in the article. The citation has now been inserted in [2 Enzymes], [2.4 Proteases and peptidases**]**, [Paragraph 1] and should read:
“[Proteases and peptidases, such as the M36 metalloprotease MepA secreted by *M. xanthus* strain DK1622, may promote the predation of prey by degrading proteins of prey cells (Berleman et al., 2014).]”
2. In the published article [Ding, Y. (2017). Isolation and Identification of Myxobacteria from the Ordos Plateau Area and Preliminary Analysis of Their Antagonistic Activity Against Phytophthora Infestans. Master, Inner Mongolia Agricultural University. https://wap.cnki.net/touch/web/Dissertation/Article/10129-1017211855.nh.html] was not cited in the article. The citation has now been inserted in [3 Small molecule compounds], [3.8 Some unknown substances], [Paragraph 1] and should read:
“[The diameters of the inhibition zones were 26 mm, 24 mm and 24 mm (Ding, 2017; Wu, 2018).]”
3. In the published article [Herrmann, J., Fayad, A.A., and Müller, R. (2017). Natural products from myxobacteria: novel metabolites and bioactivities. Natural Product Reports 34(2), 135-160. 10.1039/c6np00106h], was not cited in the article. The citation has now been inserted in [3 Small molecule compounds], [3.5 *Hyalodione***]**, [Paragraph 1] and should read:
“[Hyalodione is a novel S-methyl cyclohexadiene-dione, which belongs to the class qinone (Herrmann et al., 2017).]”
4. In the published article [Iizuka, T., Jojima, Y., Fudou, R., Hiraishi, A., Ahn, J.-W., and Yamanaka, S. (2003a). Plesiocystis pacifica gen. nov., sp. nov., a marine myxobacterium that contains dihydrogenated menaquinone, isolated from the Pacific coasts of Japan. International Journal of Systematic and Evolutionary Microbiology 53(1), 189-195. 10.1099/ijs.0.02418-0] was not cited in the article. The citation has now been inserted in [1 Introduction], [Paragraph 1] and should read:
“[Some halophilic myxobacteria i.e., *Haliangium* spp. (Ryosuke et al., 2002), *Plesiocystis pacifica* (Iizuka et al., 2003a) and *Enhygromyxa salina* (Iizuka et al., 2003b) had been isolated from marine environment.]”
5. In the published article [Iizuka, T., Jojima, Y., Fudou, R., Tokura, M., Hiraishi, A., and Yamanaka, S. (2003b). Enhygromyxa salina gen. nov., sp. nov., a Slightly Halophilic Myxobacterium Isolated from the Coastal Areas of Japan. Systematic and Applied Microbiology 26(2), 189-196. 10.1078/072320203322346038] was not cited in the article. The citation has now been inserted in [1 Introduction], [Paragraph 1] and should read:
“[Some halophilic myxobacteria i.e., *Haliangium spp*. (Ryosuke et al., 2002), *Plesiocystis pacifica* (Iizuka et al., 2003a) and *Enhygromyxa salina* (Iizuka et al., 2003b) had been isolated from marine environment.]”
6. In the published article [Kaushal, G., and Singh, S.P. (2020). Comparative genome analysis provides shreds of molecular evidence for reclassification of Leuconostoc mesenteroides MTCC 10508 as a strain of Leu. suionicum. Genomics 112(6), 4023-4031. 10.1016/j.ygeno.2020.06.040] was not cited in the article. The citation has now been inserted in [2. Enzymes], [Paragraph 1] and should read:
“[GHs hydrolyze glycosidic bonds and play an important role in the hydrolysis and synthesis of sugars and glycoconjugates in organisms (Kaushal and Singh, 2020).]”
7. In the published article [Mansfield, J., Genin, S., Magori, S., Citovsky, V., Sriariyanum, M., Ronald, P., et al. (2012). Top 10 plant pathogenic bacteria in molecular plant pathology. Molecular Plant Pathology 13(6), 614-629. 10.1111/j.1364-3703.2012.00804.x.] was not cited in the article. The citation has now been inserted in [2. Enzymes], **[**2.4 Proteases and peptidases**]**, [Paragraph 1] and should read:
“[Tomato bacterial wilt (TBW) caused by *Ralstonia solanacearum* is one of the most destructive soil-borne diseases, and tomato production has suffered huge losses due to the epidemic of TBW (Mansfield et al., 2012).]”
8. In the published article [Moeller, M., Norris, M.D., Planke, T., Cirnski, K., Herrmann, J., Müller, R., et al. (2019). Scalable Syntheses of Methoxyaspartate and Preparation of the Antibiotic Cystobactamid 861-2 and Highly Potent Derivatives. Organic Letters 21(20), 8369-8372. 10.1021/acs.orglett.9b03143] was not cited in the article. The citation has now been inserted in [3 Small molecule compounds], **[**3.4 Cystobactamid derivatives**]**, [Paragraph 1] and should read:
“[Moeller et al. (2019) compared the antibacterial properties of this class of substanaces, the cyano derivative of cystobactamide 861-2(5) had antimicrobial activity against Gram-negative bacteria and its activity was higher than that of any natural cystobactamide tested so far.]”
9. In the published article [Nickeleit, I., Zender, S., Sasse, F., Geffers, R., Brandes, G., Sörensen, I., et al. (2008). Argyrin A Reveals a Critical Role for the Tumor Suppressor Protein p27kip1 in Mediating Antitumor Activities in Response to Proteasome Inhibition. Cancer Cell 14(1), 23-35. 10.1016/j.ccr.2008.05.016] was not cited in the article. The citation has now been inserted in [3 Small molecule compounds], **[**3.6 Argyrin derivatives**]**, [Paragraph 1] and should read:
“[The culture medium of the strains of *Archangium gephyra* contained a group of cyclic peptides composed of naturally produced octapeptides, which exhibited strong antibiotic effects against *P. aeruginosa* (Nickeleit et al., 2008; Stauch et al., 2010; Wieland et al., 2022).]”
10. In the published article [Odintsov, S.G., Sabala, I., Marcyjaniak, M., and Bochtler, M. (2004). Latent LytM at 1.3Å Resolution. Journal of Molecular Biology 335(3), 775-785. 10.1016/j.jmb.2003.11.009] was not cited in the article. The citation has now been inserted in [2. Enzymes], **[**2.4 Proteases and peptidases**]**, [Paragraph 1] and should read:
“[Proteins of the M23 family were endopeptidases that cleaved bacterial cell wall peptidoglycan by degrading the peptide bonds of cross-linked peptides (Odintsov et al., 2004).]”
11. In the published article [Oren, A., and Garrity, G. M. (2021). Valid publication of the names of forty-two phyla of prokaryotes. International Journal of Systematic and Evolutionary Microbiology 71(10). 10.1099/ijsem.0.005056] was not cited in the article. The citation has now been inserted in [1 Introduction], [Paragraph 1] and should read:
“[Myxobacteria are microorganisms of the phylum Myxococcota (Waite et al., 2020; Oren and Garrity, 2021), which are well known for their complex life cycles and unique social behaviors.]”
12. In the published article [Paitan Y, Orr E, Ron EZ, and E., R. (1999). A nonessential signal peptidase II (Lsp) of Myxococcus xanthus might be involved in biosynthesis of the polyketide antibiotic TA. Journal of Bacteriology 181(18), 5644-5651. 10.1128/JB.181.18.5644-5651.1999] was not cited in the article. The citation has now been inserted in [3 Small molecule compounds], **[**3.3 Myxovirescin**]**, [Paragraph 1] and should read:
“[The antibiotic TA could inhibit the incorporation of diamibopimelic acid and uridine diphosphate-N-acetylglucosamine into *E. coli* cell wall, and antibiotic TA interfered with the polymerizaton of the lipid-disacchar-pentapeptide (Rosenberg et al., 1982; Paitan et al., 1999).]”
13. In the published article [Pogorevc, D., Tang, Y., Hoffmann, M., Zipf, G., Bernauer, H.S., Popoff, A., et al. (2019). Biosynthesis and Heterologous Production of Argyrins. ACS Synthetic Biology 8(5), 1121-1133. 10.1021/acssynbio.9b00023] was not cited in the article. The citation has now been inserted in [3 Small molecule compounds], **[**3.6 Argyrin derivatives**]**, [Paragraph 1] and should read:
“[Among them, argyrin participates in the non-ribosomal peptide synthetase pathway, combining with elongation factor G (EF-G) as its target (Pogorevc et al., 2019).]”
14. In the published article [Ren, X., Wu, Z., Cui, H., Gao, X., and Feng, F. (2016). Isolation and identification of antagonistic strain YR-7 of Phytophthora infestans and its active substances. Bulletin of Microbiology 43(07), 1513-1523. 10.13344/j.microbiol.china.150617] was not cited in the article. The citation has now been inserted in [3 Small molecule compounds], [3.8 Some unknown substances], [Paragraph 1] and should read:
“[The experimental results proved that the active substance against *P. infestans* is a non-protein substance (Ren et al., 2016).]”
15. In the published article [Rix, U., Fischer, C., Remsing, L.L., and Rohr, J.r. (2002). Modification of post-PKS tailoring steps through combinatorial biosynthesis. Natural Product Reports 19(5), 542-580. 10.1039/b103920m.] was not cited in the article. The citation has now been inserted in [1 Introduction], [Paragraph 1] and should read:
“[For example, in contrast to Actinomycetes derivatives, most small molecules of myxobacteria are not glycosylated (Rix et al., 2002).]”
16. In the published article [Rosenberg E, Fytlovitch S, Carmeli S, and Y., K. (1982). Chemical properties of Myxococcus xanthus antibiotic TA. The Journal of Antibiotics 35(7), 788-793. 10.7164/antibiotics.35.788] was not cited in the article. The citation has now been inserted in [3 Small molecule compounds], [3.3 Myxovirescin], [Paragraph 1] and should read:
“[The antibiotic TA was produced and named after M. vanthits strain TA(ATCC31046) (Rosenberg et al., 1982). The antibiotic TA could inhibit the incorporation of diamibopimelic acid and uridine diphosphate-N-acetylglucosamine into E. coli cell wall, and antibiotic TA interfered with the polymerizaton of the lipid-disacchar-pentapeptide (Rosenberg et al., 1982; Paitan et al., 1999).]”
17. In the published article [Ryosuke Fudou, Yasuko Jojima, Takashi Iizuka, and Yamanaka, S. (2002). Haliangium ochraceum gen. nov., sp. nov. and Haliangium tepidum sp. nov.: novel moderately halophilic myxobacteria isolated from coastal saline environments. The Journal of general and applied microbiology 48(2), 109-116. 10.2323/jgam.48.109] was not cited in the article. The citation has now been inserted in [1 Introduction], [Paragraph 1] and should read:
“[Some halophilic myxobacteria i.e., *Haliangium spp*. (Ryosuke et al., 2002), *Plesiocystis pacifica* (Iizuka et al., 2003a) and *Enhygromyxa salina* (Iizuka et al., 2003b) had been isolated from marine environment.]”
18. In the published article [Saggu, S.K., Nath, A., and Kumar, S. (2023). Myxobacteria: biology and bioactive secondary metabolites. Research in Microbiology 174(7), 104079. 10.1016/j.resmic.2023.104079] was not cited in the article. The citation has now been inserted in [1 Introduction], [Paragraph 1] and should read:
“[Myxobacteria have a wide range of habitats, including soil rich in organic matter, rotting wood, animal dung and marine environment (Saggu et al., 2023).]”
19. In the published article [Seef, S., Herrou, J., de Boissier, P., My, L., Brasseur, G., Robert, D., et al. (2021). A Tad-like apparatus is required for contact-dependent prey killing in predatory social bacteria. eLife 10. 10.7554/eLife.72409] was not cited in the article. The citation has now been inserted in [4 Discussion], [Paragraph 2] and should read:
“[The Tad-like system mediates the contact-dependent killing of myxobacteria on prey cells (Seef et al., 2021).]”

In the published article, there was an error in the Figure 1 and in the legend for Figure 1 as published. In the small molecule substances, we changed change mucin to myxovirescin, cyclosporamiade analogues to cystobacamids derivatives, we deleted coralmycin A hyaluronic acid dione protocystin A and enhygromic acid(1), deoxyenhygrolides A(2), deoxyenhygrolides B(3), and added hyalodione and argyrin derivatives, unknown substances. Furthermore, we changed pathogens to plant pathogens. The corrected Figure 1 and its caption appears below.

**Figure 1:**
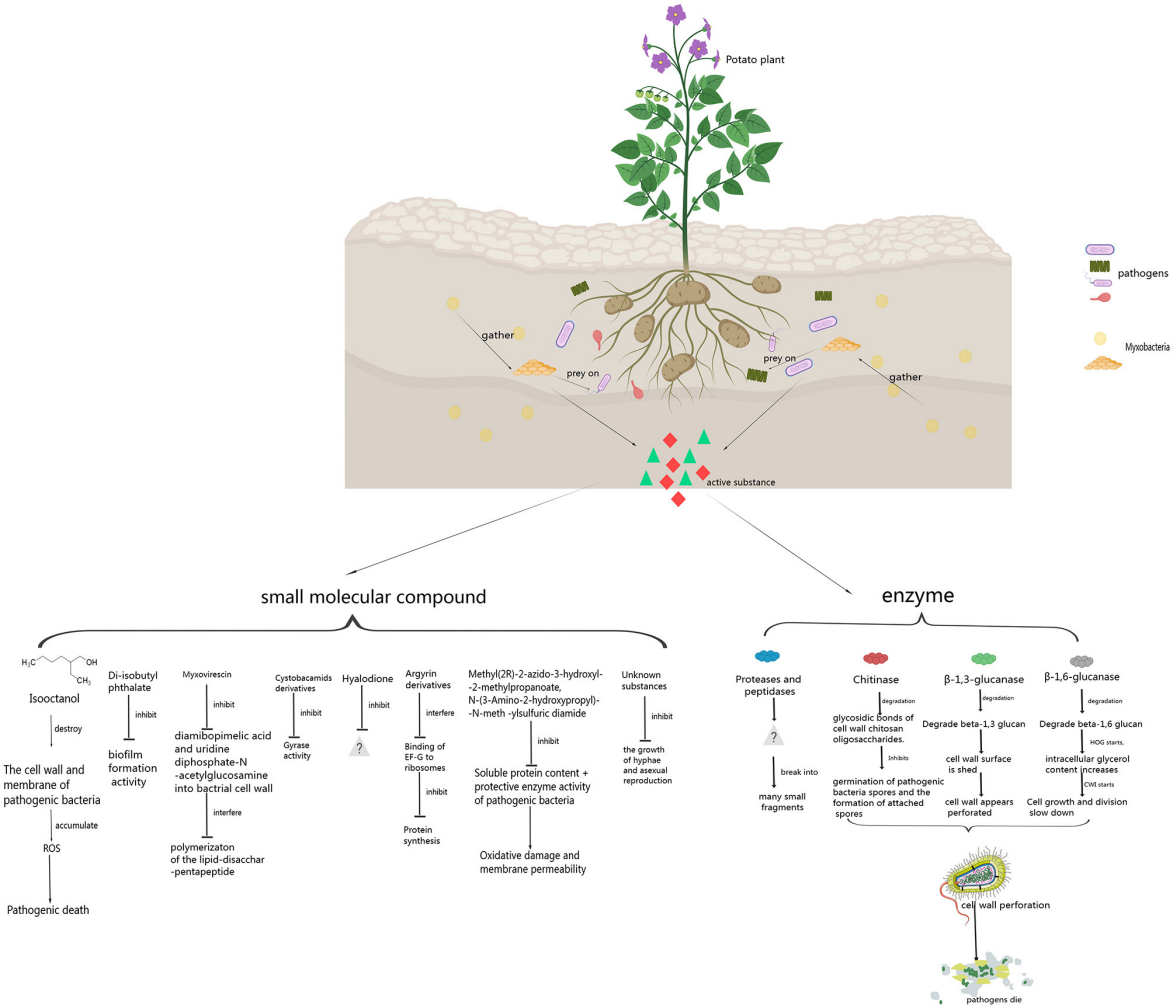
Control principle of myxobacteria against plant pathogens.

In the published article, there was an error in the Table 1 as published. Antagonistic substances are divided into two categories, namely enzymes and small molecule substances. Enzymes contain β-1,6-glucanase, β-1,3-glucanase, Chitinase, Proteases and peptidases, Formaldehyde dismutase. Small molecule compounds contain Isooctanol, Di-isobutyl phthalate, Myxovirescin, Cystobacamids derivatives, Hyalodione, Argyrin derivatives. The corrected table appears below.

**Table 1:** Control principle of myxobacteria against pathogens.

**Antagonistic substances**	**Antagonistic principle**	**References**
**Enzymes**		
β-1,6-glucanase	Degrades β-1,6-glucan. When HOG is activated, the intracellular glycerol content increases and the osmotic pressure increases; when CWI is activated, cell growth and division slow down	Zhou et al. (2019) and Ye et al. (2020a)
β-1,3-glucanase	Hydrolyzesβ-1,3-glucan. The cell wall surface falls off and a perforated structure appears in the cell wall	Zhou et al. (2019) and Zhang et al. (2023)
Chitinase	Degrades the glycosidic bonds of cell wall chitosan oligosaccharides. Inhibits the germination of pathogenic spores and the formation of attached spores	Li et al. (2019a)
Proteases and peptidases	The morphology of *R. solanacearum* changed significantly, and the cells were broken into many small fragments	Dong et al. (2023)
Formaldehyde dismutase	Formaldehyde is concerted into formate and methanol to protect myxobactria	Willsey et al. (2016) and Sutton et al. (2019)
**Small molecule compounds**		
Isooctanol	Destroy the cell wall and cell membrane of pathogenic bacteria, accumulate ROS, and cause cell death	Ye et al. (2020a)
Di-isobutyl phthalate	Exhibits biofilm formation inhibitory activity against bacteria	Sharma et al. (2021)
Myxovirescin	Inhibits the incorporation of diamibopimelic acid and uridine diphosphate-N-acetylglucosamine into bactrial cell wall, and interferes with the polymerizaton of the lipid-disacchar-pentapeptide	Rosenberg et al. (1982) and Paitan et al. (1999)
Cystobacamids derivatives	Inhibits bacterial gyrase to exert antibacterial activity	Kim et al. (2019) and Solga et al. (2024)
Hyalodione	Strongly inhibits the activity of pathogenic bacteria.	Okanya et al. (2012)
Argyrin derivatives	Participate in the non-ribosomal peptide synthase pathway and inhibit protein synthesis by interfering with the binding of elongation factor G (EF-G) to ribosomes	Pogorevc et al. (2019) and Wieland et al. (2022)
Methyl(2R)-2-azido-3-hydroxyl-2-methylpropanoate, N-(3-Amino-2-hydroxypropyl)-N-meth -ylsulfuric diamide	Reduce the content of soluble proteins and activity of protective enzymes in pathogenic bacteria, and increase oxidative damage and cell membrane permeability	Wu (2018)
Some unknown substances	Inhibits the growth of hyphae and asexual reproduction of *Phytophthora infestans*	Ren et al. (2016), Ding (2017), Wu (2018), and Zhao (2018)

In the published article, there was an error in [Table 1] as published. The corrected [Table 1 Active substances of myxobacteria against plant diseases and their action mechanisms] and its caption ^**^ [Table 1 Active substances of myxobacteria against plant diseases and their action mechanisms] appear below.

In the published article, there was **31** errors.

1. [The relevant content was added to the text, the abstract also made corresponding changes.] A correction has been made to [Abstract].

This sentence previously stated: “[Myxobacteria have a complex life cycle and unique social behavior, and obtain nutrients by preying on bacteria and fungi in soil. Chitinase, β-1,3 glucanase and β-1,6 glucanase produced by myxobacteria can degrade the glycosidic bond of cell wall of some plant pathogenic fungi, resulting in a perforated structure in the cell wall. In addition, isooctanol produced by myxobacteria can lead to the accumulation of intracellular reactive oxygen species in some pathogenic fungi and induce cell apoptosis. Myxobacteria can also perforate the cell wall of some plant pathogenic oomycetes by β-1,3 glucanase, reduce the content of intracellular soluble protein and protective enzyme activity, affect the permeability of oomycete cell membrane, and aggravate the oxidative damage of pathogen cells. Small molecule compounds such as diisobutyl phthalate and myxovirescin produced by myxobacteria can inhibit the formation of biofilm and lipoprotein of bacteria, and cystobactamids can inhibit the activity of DNA gyrase, thus changing the permeability of bacterial cell membrane. Myxobacteria, as a new natural compound resource bank, can control plant pathogenic fungi, oomycetes and bacteria by producing carbohydrate active enzymes and small molecular compounds, so it has great potential in plant disease control.]” The corrected sentence appears below: “[Myxobacteria have a complex life cycle and unique social behavior. They can prey on plant pathogenic fungi, bacteria, and oomycetes in the soil by producing some enzymes and small molecule compounds. The enzymes mainly include β-1,6-glucanase, β-1,3-glucanase, chitinase, protease, peptidase, and formaldehyde dismutase. β-1,6-glucanase, β-1,3-glucanase, and chitinase can degrade the glycosidic bonds in the cell wall of plant pathogen, causing some holes to form on the cell walls of the plant pathogen. Proteases and peptidases can break plant pathogenic cells into many small fragments and facilitate extracellular digestion of proteins during myxobacterial predation. Formaldehyde dismutase converts formaldehyde to formate and methanol, it can help myxobactria protect themselves in the process of predation. Small molecule substances produced by myxobacteria include isooctanol, di-isobutyl phthalate, myxovirescin, cystobactamid derivatives, hyalodione, argyrin derivatives, Methyl (2R)-2-azido-3-hydroxyl-2-methylpropanoate and N-(3-Amino-2-hydroxypropyl)-N-meth-ylsulfuric diamide, etc. Isooctanol destroyed the cell wall and cell membrane of plant pathogen, causing intracellular reactive oxygen species (ROS) to accumulate, leading to apoptosis and cell death. Di-isobutyl phthalate had biofilm inhibitory activity against bacteria. Myxovirescin could inhibit the incorporation of diamibopimelic acid and uridine diphosphate-N-acetylglucosamine intobacterial cell wall, and interfered with the polymerizaton of the lipid-disacchar-pentapeptide. Cystobactamid derivatives exerted their natural antibacterial properties by inhibition of bacterial gyrases. Hyalodione had broad antibacterial and antifungal activity. Argyrin derivatives inhibited protein synthesis by interfering with the binding of elongation factor G (EF-G) to ribosomes. Methyl (2R)-2-azido-3-hydroxyl-2-methylpropanoate and N-(3-Amino-2-hydroxypropyl)-N-meth-ylsulfuric diamide reduced the content of soluble proteins and the activity of protective enzymes (PPO, POD, PAL, and SOD) in plant pathogen, increased oxidative damage and cell membrane permeability. Myxobacteria, as a new natural compound resource bank, can control plant pathogenic fungi, oomycetes and bacteria by producing some enzymes and small molecule compounds, so it has great potential in plant disease control.].”

2. [There are two opinions on the species of myxobacteria, it is not yet certain which one is correct]. A correction has been made to [1 Introduction], [Paragraph 1].

This sentence previously stated: “[Myxobacteria are a type of deltaproteobacteria with rod-shaped vegetative cells (Velicer and Vos, 2009), which are well known for their complex life cycles and unique social behaviors. Myxobacteria have a wide range of life, and most of them live in soil, especially on land rich in microorganisms and organic matter. Recent studies have found that they can survive in high-salinity environments (Gemperlein et al., 2018). Research over the past few decades has proven that myxobacteria have become a resource library of new natural compounds, ranking second only to Actinomycetes and Bacillus among prokaryotes (Arguelles-Arias et al., 2009; Weissman and Müller, 2010). Metabolites produced by myxobacteria often have structures that other microbial metabolites do not have, and approximately 40% of myxobacterial metabolites have novel chemical structures. For example, in contrast to actinomycete derivatives, most small molecules of myxobacterial origin are not glycosylated (Rix et al., 2002). It iscurrently unclear why myxobacteria produce large amounts of metabolites, but researchers generally believe that metabolites play an important role in regulating cell–cell interactions within the colony (Davies et al., 2006) and in prey hunting (Xiao et al., 2011).]” The corrected sentence appears below: “[Myxobacteria are microorganisms of the phylum Myxococcota (Waite et al., 2020; Oren and Garrity, 2021), which are well known for their complex life cycles and unique social behaviors. Myxobacteria have a wide range of habitats, including soil rich in organic matter, rotting wood, animal dung and marine environment (Saggu et al., 2023). They can survive in high-salinity environments (Gemperlein et al., 2018). Some halophilic myxobacteria i.e., *Haliangium* spp. (Ryosuke et al., 2002), *Plesiocystis pacifica* (Iizuka et al., 2003a) and *Enhygromyxa salina* (Iizuka et al., 2003b) had been isolated from marine environment. Research over the past few decades has proven that myxobacteria have become a resource library of new natural compounds, ranking second only to *Actinomycetes* and *Bacillus* among prokaryotes (Arguelles-Arias et al., 2009; Weissman and Müller, 2010). Metabolites produced by myxobacteria often have structures that other microbial metabolites do not have, and ~40% of myxobacterial metabolites have novel chemical structures. For example, in contrast to *Actinomycetes* derivatives, most small molecules of myxobacteria are not glycosylated (Rix et al., 2002). It is currently unclear why myxobacteria produce large amounts of metabolites, but researchers generally believe that metabolites play an important role in regulating cell-to-cell interactions within a population (Davies et al., 2006) and in prey hunting (Xiao et al., 2011).]”

3. A correction has been made to [1 Introduction], [Paragraph 2].

This sentence previously stated: “[Myxobacteria prey on plant pathogen and destroy pathogen's cell morphology and structure. The destroyed pathogenic cells are surrounded by many filamentous substances. The cell structure is loose and irregular, and the cell contents overflow, effectively lysing the pathogenic cells. Myxobacteria produce extracellular enzymes with carbohydrate-active enzymes (CAZymes), mainly glycosyltransferases (GTs), glycoside hydrolases (GHs), carbohydrate-binding modules (CBMs) and polysaccharide (PLs), etc. CAZymes can modify the glycosidic bonds of carbohydrates and are important basic functional units in carbohydrate metabolism pathways, while GHs can degrade the glycosidic bonds of peptidoglycan in the cell wall (Dong et al., 2023). When myxobacteria prey on prey, they can kill microorganisms and lyse cells by producing metabolites such as antibiotics, cell wall degrading enzymes, lipases, nucleases, polysaccharases, and proteases, thereby clearing biological macromolecules.]” The corrected sentence appears below: “[Myxobacteria can prey on plant pathogen and destroy pathogen's cell morphology and structure. When myxobacteria prey on pathogen, they can kill microorganisms and lyse cells by producing metabolites such as antibiotics, cell wall degrading enzymes, lipases, nucleases, polysaccharases, and proteases, thereby clearing the pathogens. The destroyed pathogenic cells are surrounded by many filamentous substances. The cell structure become loose and irregular, and the cell contents overflow, and eventually the pathogen lyse and die.]”

4. A correction has been made to [1 Introduction], [Paragraph 3].

This sentence previously stated: “[Myxobacteria are abundant in surface soils, but only a few studies have reported their role in agricultural soils. Myxobacteria are potential biocontrol agents. The application of biological control agents (BCAs) in agricultural planting can reduce the use of pesticides, reduce the adverse effects caused by excessive use of chemicals and achieve the purpose of controlling soil-borne plant diseases. BCAs are very effective in preventing and managing plant diseases, achieve ecological and economic benefits such as increasing agricultural output and reducing environmental pollution. Myxobacteria are potential BCAs, but their mode of action has been less studied at the molecular level. Their research can provide new potential ways for biological control of plant diseases and insect pests.]” The corrected sentence appears below: “[Therefore, myxobacteria can serve as biological control agents (BCAs) of plant diseases (Ye et al., 2020b). The BCAs in agricultural planting can reduce the use of pesticides, reduce the adverse effects caused by excessive use of chemicals and achieve the purpose of controlling soil-borne plant diseases. The BCAs are very effective in preventing and managing plant diseases and achieving ecological and economic benefits such as increasing agricultural output and reducing environmental pollution. Research on myxobacteria can provide new potential ways for biological control of plant diseases. This paper reviews the research progress on the active substances of myxobacteria against plant diseases and their action mechanisms.]”

5. A correction has been made to [2 Enzymes], [Paragraph 1].

This sentence previously stated: “[At present, the mechanism by which myxobacteria inhibit the growth of pathogenic bacteria has not been thoroughly studied. Some studies have only been conducted to the extent that myxobacteria inhibit the growth of plant pathogen. There is still great research value and space in the future. Research on the active enzymes of myxobacteria that prey on plant pathogens is mainly divided into three categories: β-1,6-glycosidase, β-1,3-glycosidase and chitinase. In addition, there are a few studies on Proteases, lipases and formaldehyde dismutase. Active enzymes produced by myxobacteria to control plant pathogens are shown in Figure 1 and Table 1.]” The corrected sentence appears below: “[Myxobacteria produce some enzymes playing important roles in preying on pathogens. These enzymes include carbohydrate-active enzymes (CAZymes), peptidases, lipases, etc. CAZymes include glycosyltransferases (GTs), glycoside hydrolases (GHs), carbohydrate esterases (CEs), auxiliary activities (AAs), carbohydrate-binding modules (CBMs), and polysaccharide lyases (PLs). CAZymes can modify the glycosidic bonds of carbohydrates and are important basic functional units in carbohydrate metabolism pathways (Dong et al., 2023). GHs hydrolyze glycosidic bonds and play an important role in the hydrolysis and synthesis of sugars and glycoconjugates in organisms (Kaushal and Singh, 2020). The enzymes produced by myxobacteria to control plant pathogens are shown in Figure 1 and Table 1.]”

6. A correction has been made to [2 Enzymes], [2.1 β-1,6-glucanase], [Paragraph 1].

This sentence previously stated: “[β-1,6-Glucan is a component of the fungal cell wall that is smaller than chitin and β-1,3-glucan and can cross-link cell wall proteins to the chitin layer and β-1, 3-Glucan layer. Inhibiting the synthesis of β-1,6-glucan is conducive to the effective disintegration and further degradation of the cell wall during the process of myxobacteria preying on plant pathogen. Coralococcus EGB-derived β-1,6-glucanase GluM is a novel family of outer membrane β-barrel proteins that can inhibit fungal embryonic tube development. β-1,6-Glucanase GluM preys on it is essential in the initial sensing and efficient decomposition of fungi and can also inhibit the growth of oomycetes.]” The corrected sentence appears below: “[β-1,6-glucan is a component of the fungal cell wall smaller than chitin and β-1,3-glucan. It can cross-link cell wall proteins to the chitin layer and β-1,3-glucan layer. Inhibiting the synthesis of β-1,6-glucan is conducive to the effective disintegration and further degradation of pathogen cell wall during the process of myxobacteria preying on plant pathogen. β-1,6-glucanase can hydrolyze the glycosidic bonds of β-1,6-glucan, thereby destroying the entire cell wall structure of fungi. β-1,6-glucanase GluM from the strain EGB of *Coralococcus* sp. is a novel family of outer membrane β-barrel proteins that can inhibit fungal embryonic tube development (Li et al., 2019b). β-1,6-glucanase GluM is essential in the initial sensing and efficient decomposition of fungi.]”

7. A correction has been made to [2 Enzymes], [2.1 β-1,6-glucanase], [Paragraph 2].

This sentence previously stated: “[Electron microscopy of the hyphae of Magnaporthe oryzae treated with β-1,6-glucanase GluM showed that the hyphae were stretched and partially broken, and the hyphal cell wall changed from a dense structure to a loose structure. The spore folds of the treated rice blast fungus were irregular, the density decreased, and the spore morphology showed a deformed state. The morphological and structural changes of M. oryzae are speculated to be due to the hydrolysis of the cell wall by β-1,6-glucanase GluM, resulting in incomplete cell structure and outflow of contents, ultimately leading to morphological changes. β-1,6-glucanase GluM limits the infection of rice by digesting the cell wall of M. oryzae (Zhou et al., 2019).]” The corrected sentence appears below: “[Electron microscopy observation of the hyphae of *Magnaporthe oryzae* treated with β-1,6-glucanase GluM showed that the hyphae were stretched and partially broken, and the hyphal cell wall changed from a dense structure to a loose structure. The spore folds of the treated *M. oryzae* were irregular. The density of spore decreased, and the morphology of spore showed a deformed state. The morphological and structural changes of *M. oryzae* were speculated to be due to the hydrolysis of the cell wall by β-1,6-glucanase GluM, resulting in incomplete cell structure and outflow of contents, ultimately leading to morphological changes. Therefore, β-1,6-glucanase GluM inhibited the infection of *M. oryzae* in rice by digesting the pathogen's cell wall (Zhou et al., 2019).]”

8. A correction has been made to [2 Enzymes], [2.1 β-1,6-glucanase], [Paragraph 3].

This sentence previously stated: “[After GluM treated the hyphae and spores of F. oxysporumf. sp. cucumerinum (FOC), there was obvious shrinkage. The cell wall of FOC appears to be perforated and damaged, the structure is loose, and large vacuoles are formed in the cells. The high osmolarity glycerol (HOG) is activated, the phosphorylation level of Hog1-likemitogen-activated proteinkinase (MAPK) in FOC cells increases significantly, and the intracellular glycerol content increases 2.6 times. The osmotic pressure in FOC cells increased, which accelerated cell lysis. The cell wall integrity (CWI) initiates the transcription of genes related to the synthesis of cell wall components, chitin synthase genes (FOX-04163), chitinase genes (FOXG-19879, FOXG-19525, FOXG-12882), the transcript levels of β-glucanase gene (FOXG-03928) and β-glucan synthase gene (FOXG-03721) increased. The transcription levels of genes related to components corresponding to cell wall integrity (FOXG-09228), programmed cell death control protein (FOXG-03587) and cell division control protein (FOXG-00362) increased, slowing down the growth and division rate of cells and activating cell apoptosis. After inoculation of Coralococcus EGB into cucumbers, it can adapt well to the soil environment, effectively reduce the abundance of soil-borne F. oxysporum, and significantly reduce the occurrence of cucumber wilt disease (Ye et al., 2020b).]” The corrected sentence appears below: “[After treated with GluM, the hyphae and spores of *Fusarium oxysporum* f. sp. *cucumerinum* (FOC) shrank obviously. The cell wall of FOC appeared to be perforated and damaged, the cell wall structure was loose, and large vacuoles formed in the cells. The High Osmolarity Glycerol (HOG) in FOC cells was activated. The phosphorylation level of Hog1-likemitogen-activated proteinkinase (MAPK) was significantly increased and the glycerol content increased 2.6 times. The osmotic pressure in FOC cells increased, which accelerated cell lysis. When the strain EGB of *Corallococcus* sp. were inoculated with the potted cucumbers, the strain could adapt well to the soil environment and effectively reduced the abundance of soil-borne *F. oxysporum* and the occurrence of cucumber wilt disease (Ye et al., 2020b).]”

9. A correction has been made to [2 Enzymes], [2.1 β-1,6-glucanase], [Paragraph 4].

This sentence previously stated: “[In the GluM transgenic experiment, the β-1,6-glucanase gene GluM was transferred into japonica rice ZH11 to obtain transgenic japonica rice with overexpression of GluM. In the fungal disease resistance experiment, the rice blast area of GluM transgenic rice was reduced by 82.7%, the sheath blight disease was reduced by 35.76–43.67%, and the incidence of rice smut disease was reduced by 65.79%. The results show that transgenic rice containing GluM protein can degrade fungal cell walls through specific hydrolysis and enhance resistance to fungal diseases (Shen et al., 2023).]” The corrected sentence appears below: “[In the *GluM* transgenic experiment, the β-1,6-glucanase gene was transferred into japonica rice variety ZH11 to obtain transgenic japonica rice with overexpression of *GluM*. In the fungal disease resistance experiment, the rice blast area of *GluM* transgenic rice was reduced by 82.7%. The sheath blight disease was reduced by 35.76%−43.67% and the incidence of rice smut disease was reduced by 65.79%. The results showed that transgenic rice containing GluM protein could degrade fungal cell walls through specific hydrolysis and enhanced resistance to fungal diseases (Shen et al., 2023).]”

10. A correction has been made to [2 Enzymes], [2.1 β-1,6-glucanase], [Paragraph 5].

This sentence previously stated: “[The β-1,6-glucanase produced by myxobacteria not only inhibits the growth of fungal pathogens, but also plays a certain role in oomycetes. The fermentation product of Coralococcus coralliformis CMC0606 has a strong inhibitory effect on Phytophthora capsici, and the diameter of its inhibition zone is 16mm (Bader et al., 2022). Coralococcus EGB has a good inhibitory effect on the growth of P. capsici, which is mainly manifested by the collapse of mycelium and the growth is obviously inhibited. Preliminary research on the disease resistance mechanism of strain EGB suggests that there may be an antibacterial protein of β-1,6-glucanase in the extracellular fermentation supernatant of EGB (Zhang et al., 2023).]” The corrected sentence appears below: “[The β-1,6-glucanase produced by myxobacteria inhibits not only the growth of pathogenic fungus, but also the growth of oomycetes. The fermentation products of *C. coralliformis* strain CMC0606 had a strong inhibitory effect on *Phytophthora capsici*, and the diameter of inhibition zone was 16 mm (Bader et al., 2022). The strain EGB had a strong inhibitory effect on the growth of *P. capsica*. The mycelium of *P. capsica* collapsed and the growth of the pathogen was obviously inhibited. The results showed that β-1,6-glucanase in the fermentation supernation of strain EGB was effective in inhibiting oomycetes (Zhang et al., 2023).]”

11. A correction has been made to [2 Enzymes], [2.2 β-1,3-glucanase], [Paragraph 1].

This sentence previously stated: “[β-1,3-Glucan is the most abundant component of the fungal cell wall. Extensive hydrolysis of fungal cell wall polymer chains by β-1,3-glucanase can reduce the mechanical strength of the cell wall, leading to the final lysis of the fungal cell. β-1,3-glucanase IamC derived from Coralococcus EGB can cleave β-1,3- or β-1,6-glucan substrates by exo-hydrolysis and hydrolyze β by endo-hydrolysis.−1,4-glucan or xylan substrate is a type of glucanase with multifunctional activity. Cu2+, Co2+, Mg2+, and Cr3+ inhibit the activity of IamC, while Mn2+ is an effective activator of IamC, indicating that IamC is a metal ion-dependent hydrolase. After exposure of M. oryzae to IamC, the germ tube and appressorium formation rates were significantly reduced from 94 and 97% to 59 and 51%. The hyphae are enlarged and deformed, and more granular contents appear inside the hyphae. A large amount of reactive oxygen species (ROS) accumulation and changes in chitin distribution can be observed in the spores and hyphae of M. oryzae. The β-1,3-glucanase IamC derived from Coralococcus EGB acts on different β-glycosidic bonds in the cell wall of M. oryzae. It is hydrolyzed from different sites of cell wall polysaccharides to exert solubilization and antibacterial activity against M. oryzae (Zhou et al., 2019).]” The corrected sentence appears below: “[β-1,3-glucan is a component of the fungal cell wall. Extensive hydrolysis of fungal cell wall polymer chains by β-1,3-glucanase can reduce the mechanical strength of the cell wall, leading to the final lysis of the fungal cell. β-1,3-glucanase IamC from strain EGB can cleave β-1,3- or β-1,6-glucan substrates by exo-hydrolysis. Cu^2+^, Co^2+^, Mg^2+^, and Cr^3+^ inhibit the activity of IamC, while Mn^2+^ is an effective activator of IamC, indicating that IamC is a metal ion-dependent hydrolase. After exposure of *M. oryzae* to IamC, the germ tube and appressorium formation rates were significantly reduced from 94 and 97% to 59 and 51%. The hyphae of *M. oryzae* was enlarged and deformed, and more granular contents appeared inside the hyphae. There was a large accumulation of reactive oxygen species (ROS) in the spores and hyphae of *M. oryzae* and the distribution of chitin in the cell wall of pathogen changed. The β-1,3-glucanase IamC derived from strain EGB acted on different β-glycosidic bonds in the cell wall of *M. oryzae*. β-glycosidic bonds of polysaccharides from different sites of cell wall of pathogen were hydrolyzed, ultimately leading to cell lysis of *M. oryzae* (Zhou et al., 2019).]”

12. A correction has been made to [2 Enzymes], [2.2 β-1,3-glucanase], [Paragraph 2].

This sentence previously stated: “[Archangium AC19 showed strong predatory activity against Phytophthora sojae P6497 and protected soybeans from stem rot. Strain AC19 was observed to prey on P. sojae, and the hyphal cell wall of P. sojae P6497 showed perforation. The results of the indoor biological control test of strain AC19 showed that strain AC19 showed significant biological control effect on soybean hypocotyls, and the supernatant significantly reduced the growth of P. sojae and inhibited infection. Experimental results show that strain AC19 is a myxobacteria that can secrete CAZymes that inhibit P. sojae. The active substance that preys on and digests P. sojae is a cell wall hydrolase. The cell wall-acting CAZymes in strain AC19 are specialized β-1,3-glucanases (AcGlu13.1, −13.2, and−13.3) that target β-1,3-glucans from Phytophthora spp. AcGlu13.1 causes cell wall surface shedding, showing degradative activity. AcGlu13.2 and AcGlu13.3 can cause perforated structures in the cell wall (Zhang et al., 2023).]” The corrected sentence appears below: “[*Archangium* strain AC19 showed strong predatory activity against *Phytophthora sojae* P6497 and protected soybeans from stem rot disease. Strain AC19 was observed to prey on *P. sojae*, and the hyphal cell wall of *P. sojae* P6497 showed perforation. The fermentation supernatant of strain AC19 significantly inhibited the growth and infection of *P. sojae*. The active substances that digested *P. sojae* were the CAZymes secreted by strain AC19. The cell wall-acting CAZymes in strain AC19 were specialized β-1,3-glucanases (AcGlu13.1,−13.2, and−13.3). These β-1,3-glucanases targeted β-1,3-glucan from the cell wall of *Phytophthora*. AcGlu13.1 caused cell wall surface shedding through its degradative activity. AcGlu13.2 and AcGlu13.3 could cause perforated structures in the cell wall (Zhang et al., 2023).]”

13. A correction has been made to [2 Enzymes], [2.3 Chitinase], [Paragraph 1].

This sentence previously stated: “[β-1,6-glucan, and chitin accounts for 22–40% of the fungal cell wall (Bowman and Free, 2006). Chitinase can hydrolyze chitin in fungal cell walls, so chitinase is regarded as an antifungal factor for biocontrol of fungal diseases and has great potential in the enzymatic synthesis and hydrolysis of chitin (Shehata et al., 2018). Coraliococcus EGB can synthesize the endochitinase CcCti1, which belongs to the glycoside hydrolase family 18 (GH18) and has potential antifungal activity. CcCti1 can not only degrade chitosan oligosaccharide, but also hydrolyze chitin into N-acetylated chitohexaose (GlcNAc)6. CcCti1 has biological control activity against the plant pathogen Magnaporthe oryzae, inhibiting the germination of conidia and the formation of appressoria of M. oryzae at a concentration of 0.08 mg/mL (Li et al., 2019a). Rice blast caused by M. oryzae is the main limiting factor in global rice production and one of the most destructive diseases in cultivated rice in the world (Talbot, 2003). Experiments on resistance to rice blast fungus have shown that transgenic plants with chitinase genes show strong resistance to rice blast. The reason may be that fungi have significant internal expansion pressure, and slight changes in cell wall integrity can cause lysis of fungal cells (Selitrennikoff, 2001). Therefore, transgenic plants with chitinase genes are more resistant to the rice blast fungus.]” The corrected sentence appears below: “[The fungal cell wall is mainly composed of chitin, β-1,3-glucan and β-1,6-glucan. The chitin accounts for 22%−40% of the fungal cell wall (Bowman and Free, 2006). Chitinase of GHs family can hydrolyze chitin in fungal cell walls, so chitinase is regarded as an antifungal factor for biocontrol of fungal diseases (Shehata et al., 2018; Li et al., 2019a). Strain EGB can synthesize the endo-chitinase CcCti1 which belongs to the GHs family 18 (GH18) and has potential antifungal activity. CcCti1 can not only degrade chitosan oligosaccharide, but also hydrolyze chitin into N-acetylated chitohexaose (GlcNAc)6. CcCti1 had biological control activity against the plant pathogen *M. oryzae*, inhibiting the germination of conidia and the formation of appressoria of *M. oryzae* at a concentration of 0.08 mg/mL (Li et al., 2019a). Rice blast caused by *M. oryzae* is the main limiting factor in global rice production and is one of the most destructive diseases in cultivated rice in the world (Talbot, 2003). The transgenic plants with chitinase genes showed strong resistance to rice blast. The reason may be that the cell wall integrity of pathogen changed, leading to significant internal expansion pressure and lysis of fungal cells (Selitrennikoff, 2001). Therefore, transgenic plants with chitinase genes were more resistant to the *M. oryzae*.]”

14. A correction has been made to [2 Enzymes], [2.4 Proteases and peptidases], [Paragraph 1].

This sentence previously stated: “[Tomato bacterial wilt (TBW) caused by Ralstonia solanacearum is one of the most destructive soil-borne diseases, and tomato production has suffered huge losses due to the epidemic of TBW (Mansfield et al., 2012). M. xanthus R31 has good biological control potential against TBW, and the biocontrol efficiency against TBW in pot experiments was as high as 81.9%. In the experiment of preying on R. solanacearum, M. xanthus GDMCC801043 appeared to prey on the bacteria on the third day. In a pot experiment on biological control of R. solanacearum, the incidence rate dropped to 38.8%, the disease index dropped to 18.0%, and the biocontrol efficiency reached 81.9%. When strain CDMCC80143 preyed on R. solanacearum, the morphology of R. solanacearum changed significantly, and the cells were broken into many small fragments. Myxobacteria secrete a large amount of filamentous extracellular substances that surround R. solanacearum. Experiments have shown that the extracellular substances may be proteases, cellulases and lipases.]” The corrected sentence appears below: “[Tomato bacterial wilt (TBW) caused by *Ralstonia solanacearum* is one of the most destructive soil-borne diseases, and tomato production has suffered huge losses due to the epidemic of TBW (Mansfield et al., 2012). *M. xanthus* R31 had good biological control potential against TBW, and the biocontrol efficiency against TBW in pot experiments was as high as 81.9%. The MEROPS database of strain R31 genome had annotated 274 proteins, including 132 metalloproteases and 107 serine proteases. Three M36 metalloproteases were identified in the R31 genome that may contribute to the extracellular digestion of proteins during predatory behavior (Dong et al., 2023). Proteins of the M23 family were endopeptidases that cleaved bacterial cell wall peptidoglycan by degrading the peptide bonds of cross-linked peptides (Odintsov et al., 2004). Proteases and peptidases, such as the M36 metalloprotease MepA secreted by *M. xanthus* strain DK1622, may promote the predation of prey by degrading proteins of prey cells (Berleman et al., 2014).]”

15. A correction has been made to [2 Enzymes], [2.5 Formaldehyde dismutase], [Paragraph 1].

This sentence previously stated: “[When Pseudomonas aeruginosa is captured, it secretes toxic formaldehyde to resist predation and produces formaldehyde detoxifying enzymes, such as formaldehyde dismutase (Fdm). Therefore, myxobacteria that have the ability to prey on P. aeruginosawill also protect themselves by producing Fdm to achieve the purpose of preying on P. aeruginosa.]” The corrected sentence appears below: “[When *Pseudomonas aeruginosa* was preyed on, it secreted toxic formaldehyde to resist predation. Fomaldehyde can be converted into formate and methanol by formaldehyde detoxifying enzymes, such as formaldehyde dismutase (Fdm), produced by *P. aeruginosa* to protect itself (Willsey et al., 2016). It was shown that myxobacteria could produce formaldehyde dismutase. Therefore, myxobacteria had the ability to prey on *P. aeruginosa* by converting toxic formaldehyde secreted by *P. aeruginosa* into non-toxic substances (Sutton et al., 2019). Myxobacteria may also be able to prey on similar plant pathogens by a similar way.]”

16. A correction has been made to [3 Small molecule compounds], [2.5 Formaldehyde dismutase], [Paragraph 1].

This sentence previously stated: “[The biological control activity of myxobacteria against pathogenic bacteria is not only active extracellular protease, but also produce volatile compounds (VOCs). These substances include isooctanol, diisobutyl phthalate, mucin, cystobactamide analogues, coral A, hyalurondione, protosin A, methyl (2R)-2-azido-3-hydroxyl-2-methylpropanoate and N-(3-amino-2-hydroxypropyl)-N-meth-ylsulfuric diamide. Small molecule compounds produced by myxobacteria to control plant pathogens are shown in Figure 1 and Table 1.]” The corrected sentence appears below: “[The biological control activity of myxobacteria against pathogen depends on not only enzymes, but also some small molecule compounds. These compounds include isooctanol, di-isobutyl phthalate, myxovirescin, cystobactamid derivatives, hyalodione, argyrin derivatives, Methyl (2R)-2-azido-3-hydroxyl-2-methylpropanoate, and N-(3-Amino-2-hydroxypropyl)-N-meth-ylsulfuric diamide, etc. Some small molecule compounds produced by myxobacteria to control plant pathogens are shown in Figure 1 and Table 1.]”

17. A correction has been made to [3 Small molecule compounds], [3.1 Isooctanol], [Paragraph 1].

This sentence previously stated: “[Coraliococcus EGB exhibits superior biological control activity against F. oxysporum. F. oxysporum is a ubiquitous soil-borne plant pathogen that can cause vascular wilt in a variety of crops (Pietro et al., 2003). A total of 32 volatile compounds produced by strain EGB were identified, and isooctanol had the highest antifungal activity. Isooctanol destroys the cell wall and cell membrane of F. oxysporum, causing intracellular reactive oxygen species (ROS) to accumulate, leading to apoptosis and cell death. A dose of only 3.75μL/plate of isooctanol is sufficient to inhibit F. oxysporum. VOCs are also suitable for controlling plant pathogens during the postharvest storage stage because VOCs are biodegradable and have no toxic residues on the product surface (Ye et al., 2020a).]” The corrected sentence appears below: “[Strain EGB exhibited superior biological control activity against *F. oxysporum. F. oxysporum* is a ubiquitous soil-borne plant pathogen that can cause vascular wilt in a variety of crops (Pietro et al., 2003). A total of 32 volatile compounds produced by strain EGB were identified, and isooctanol had the highest antifungal activity. The mycelia of *F. oxysporum* treated with isooctanol showed severely shrinkage and collapse. The hyphae of *F. oxysporum* treated by isooctanol, the transcript levels of many genes related to the cell wall integrity (CWI) pathway and redox reactions were significantly increased by 15-to 40-fold. The transcription levels of chitin synthase (FOXG_12345, FOXG_10443 and FOXG_04179), chitinase (FOXG_19879 and FOXG_17332), endo-1,3 (4)-β-glucanase (FOXG_22849, FOXG_10637 and FOXG_03928) were upregulated after the mycelia of *F. oxysporum* were treated with isooctanol. The transcription levels of genes related to components corresponding to cell wall integrity (FOXG_09228), programmed cell death control protein (FOXG_03587) and cell division control protein (FOXG_00362) increased, slowing down the growth and division rate of cells, and activating cell apoptosis. Isooctanol destroyed the cell wall and cell membrane of *F. oxysporum*, causing intracellular reactive oxygen species (ROS) to accumulate, leading to apoptosis and cell death. A dose of only 3.75 μL/plate of isooctanol was sufficient to inhibit *F. oxysporum* (Ye et al., 2020a).]”

18. A correction has been made to [3 Small molecule compounds], [3.2 Isooctanol], [Paragraph 1].

This sentence previously stated: “[Myxobacterium strain ST/P/71 has obvious antibacterial activity against Bacillus subtilis, and the ST/P/71 extract shows inhibition against B. subtilis. The secondary metabolites of strain ST/P/71 were separated and purified, and two pure compounds were obtained at RT54.24 (Ra2) and RT71.27 (Ra3). Ra2 was identified as diisobutyl phthalate. This substance shows biofilm formation inhibitory activity against B. subtilis, with an MBIC50 of 2.702μg/mL. Currently, there are few studies on myxobacteria inhibiting bacterial biofilm formation (Sharma et al., 2021).]” The corrected sentence appears below: “[*M. fulvus* strain ST/P/71 had obvious antibacterial activity against *B. subtilis*, and the extract from the strain ST/P/71 mainly showed inhibition activity against *B. subtilis*. During seperating by Reverse Phase High Performance Liquid Chromatography (RP-HPLC), two pure compounds were eluted at RT 54.24 (Ra2) and RT 71.27 (Ra3). Ra2 was identified as di-isobutyl phthalate. This substance showed biofilm formation inhibitory activity against *B. subtilis*, with an MBIC_50_ of 2.703 μg/mL (Sharma et al., 2021). Di-isobutyl phthalate had biofilm formation inhibitory activity against *B. subtilis*, so it could have similar functions to plant pathogenic bacteria. Therefore, di-isobutyl phthalate may have great potential in the prevention and control of plant pathogenic bacteria.]”

19. A correction has been made to [3 Small molecule compounds], [3.2 Isooctanol], [Paragraph 2], delete the paragraph.
20. A correction has been made to [3 Small molecule compounds], [3.3 Myxovirescin], [Paragraph 1].

This sentence previously stated: “[Bacterial biofilms protect microbial communities from predation, and B. subtilis biofilm formation is triggered by thiopeptide antibiotics. Soil bacteria interact with each other in a competitive and cooperative manner. Most bacteria tested under laboratory conditions are unable to resist Myxococcus xanthus, but Bacillus subtilis NCIB3610 (Zeigler et al., 2008) can temporarily resist the predation of M. xanthus. This short-term protection Special metabolite protection is required (Müller et al., 2014). One of the 18 metabolites of M. xanthus DK1622 is mucin, which has antibacterial activity and can inhibit the production of lipoproteins to meet the needs of preying on bacteria (Xiao et al., 2011).]” The corrected sentence appears below: “[One of the 18 metabolites of *M. xanthus* strain DK1622 is polyketide myxovirescin (antibiotic TA), which had antibacterial activity. Myxovirescin played an important role in killing *Escherichia coli*, including lysis and subsequent predation (Xiao et al., 2011). The antibiotic TA was produced and named after *M. vanthits* strain TA(ATCC31046) (Rosenberg et al., 1982). The antibiotic TA could inhibit the incorporation of diamibopimelic acid and uridine diphosphate-N-acetylglucosamine into *E. coli* cell wall, and antibiotic TA interfered with the polymerizaton of the lipid-disacchar-pentapeptide (Rosenberg et al., 1982; Paitan et al., 1999). Myxobacteria encode a variety of substances to attack prey cells, with antibiotics serving as a front line weapon. Antibiotics can act as small molecule weapons to penetrate and kill or neutralize the metabolism of prey. The prevention and control principle of myxovirescin against bacterial pathogens has been relatively clear. This substance can be applied to research on prevention and control of plant pathogenic bacteria.]”

21. A correction has been made to [3 Small molecule compounds], [3.4 Cystobactamid derivatives], [Paragraph 1].

Divide “[Myxobacteria produce specialized metabolites when antimicrobial and exhibit cooperative, swarming predatory strategies, and the production of specialized metabolites and lytic proteins of myxobacteria is related to their predation (Akbar and Stevens, 2021). During the biological screening process of new myxobacterial extracts, substances that inhibit Pseudomonas aeruginosa were obtained. The target compound has a UV absorption spectrum similar to cystobactamide (Hüttel et al., 2017). Seven new coralmycin derivatives and three known compounds were isolated from the culture of C. coralline M23. The coralmycin derivatives are C (1), D (2), E (3), and F (4), G (5), H (6) and I (7), the known compounds are cystobactamide 891-2 (8), 905-2 (9), and 507 (10). Based on the structure–activity relationship of the antibacterial and DNA gyrase inhibitory activities of the obtained compound, the principle of its action in inhibiting Pseudomonas was analyzed. The structure of p-nitrobenzoic acid is crucial for inhibiting DNA gyrase and the growth of bacteria, while the nitro part of p-nitrobenzoic acid and the C-4 position of the nitro part of the isopropyl chain are important for certain Gram-negative bacteria. The permeability of bacteria (e.g., Pseudomonas aeruginosa, Klebsiella pneumoniae) is of great significance. The β-methoxyasparagine structure may affect bacterial cellular prey uptake (Kim et al., 2019). Another culture isolation of strain M23 also yielded coralmycin A (1), B (2) and another derivative, cystobactin 919-2. Coralmycin A has the strongest antibacterial activity against Gram-negative bacteria and has a wide antibacterial spectrum. Research data shows that the hydroxyl groups of the β-methoxyasparagine unit and the benzoic acid unit are extremely important for antibacterial activity (Kim et al., 2016).]” into 2 paragraphs, the first paragraph is “[Myxobacteria produce specialized metabolites when preying, the production of specialized metabolites and lytic proteins of myxobacteria is related to their predation (Akbar and Stevens, 2021). Müller et al. found that the target compound inhibiting *P. aeruginosa* and other bacterial pathogens had a UV absorption spectrum similar to that of cystobactamides. Cystobacamids are aromatic oligoamides that exert their natural antibacterial properties by inhibition of bacterial gyrases (Solga et al., 2024). The improved orthogonally functionalized methoxyaspartate of cystobactamides could expand the synthesis of new cystobactamides. At present, four new types of cystobactamides 919-1 (1), 919-2 (2), 920-1 (3), 920-2 (4) and cystobactamides 861-2 (5) had been successfully synthesized and measured. Moller et al. (2019) compared the antibacterial properties of this class of substanaces, the cyano derivative of cystobactamide 861-2(5) had antimicrobial activity against Gram-negative bacteria and its activity was higher than that of any natural cystobactamide tested so far.].” The second paragraph is “[Culture isolation of *C. coralline* strain M23 yielded coralmycin A (1), B (2) and another derivative, cystobactin 919-2. Coralmycin A had the strongest antibacterial activity against Gram-negative bacteria and had a wide antibacterial spectrum. Coralmycin A and B were cystobactamid derivatives (Kim et al., 2016). Seven new coralmycin derivatives and three known compounds were also isolated from the another culture of strain M23. The coralmycin derivatives are C (1), D (2), E (3), F (4), G (5), H (6), and I (7), the known compounds are cystobactamide 891-2(8), 905-2(9), and 507(10). The compounds had DNA gyrase inhibitory activity and antibacterial activity. The β-methoxyasparagine structure of coralmycin may affect prey ingestion (Kim et al., 2019). Research on the structure of cystobactamides can be used to develop new structural substances with more antibacterial activity. Therefore, this type of substances may also have great potential in the prevention and control of Gram-negative plant pathogenic bacteria.]”

22. A correction has been made to [3 Small molecule compounds], [3.5 Hyalodione], [Paragraph 1].

This sentence previously stated: “[Myxobacteria isolated from Indian soil samples, the extracts of Coralococcus parvum S104 and S145 showed broad-spectrum antibacterial activity. The WE and DE of GNDU172 showed obvious activity against B. subtilis, and the DE of strain S213 and strain S223 showed similar activity. Antibacterial activity spectrum, active against Pseudomonas syringae, while DE of S223 has strong activity against B. cereus (Kumar et al., 2017). Hyalodione was isolated from the extract of the myxobacterium hyalangiumminutum NOCB-2T and has antibacterial activity against Pseudomonas aeruginosa with a MIC value of 8.5μg/mL (Okanya et al., 2012).]” The corrected sentence appears below: “[Hyalodione isolated from the extract of the *Hyalangium minutum* strain NOCB-2T had antibacterial activity against *P. aeruginosa*. Hyalodione is a novel S-methyl cyclohexadiene-dione, which belongs to the class qinone (Herrmann et al., 2017). Hyaladione had broad antibacterial and antifungal activity. The tested strains included *P. aeruginosa, Rhodotorula glutarum* and *Staphylococcus aureus* (Okanya et al., 2012). Research on how hyalodione control bacteria and fungi need to be continued. Hyalodione also has great potential in controlling plant pathogenic bacteria and fungi.]”

23. A correction has been made to [3 Small molecule compounds], [3.6 Argyrin derivatives], [Paragraph 1]. This sentence previously stated: “[The culture medium of Archangiumgephyra strains contains a group of cyclic peptides composed of 8 amino acid residues, namely protocystins A-H, which exhibit good antibiotic effects against Pseudomonas aeruginosa (Stauch et al., 2010). Among them, protocystin A combines with elongation factor G (EF-G) as its target. Several derivatives of protocystin A can be obtained by modifying the methoxytryptophan residue, and the methoxy group is located the antibacterial activity will be lost if the position is replaced by other substituents (Siebert et al., 2019).]” The corrected sentence appears below: “[The culture medium of the strains of *Archangium gephyra* contained a group of cyclic peptides composed of naturally produced octapeptides, which exhibited strong antibiotic effects against *P. aeruginosa* (Nickeleit et al., 2008; Stauch et al., 2010; Wieland et al., 2022). Among them, argyrin participates in the non-ribosomal peptide synthetase pathway, combining with elongation factor G (EF-G) as its target (Pogorevc et al., 2019). Argyrins inhibit protein synthesis by interfering with EF-G binding to the ribosome (Wieland et al., 2022). Several derivatives of argyrin A could be obtained by modifying the methoxytry ptophan residue. The methoxy group of this residue was crucial for its antibacterial activity and its activity will be lost if the position is replaced by other substituents (Siebert et al., 2019). Argyrin has antibacterial activity against bacteria, so there is great practical value to study its antibacterial activity against plant pathogenic bacteria.]”
24. A correction has been made to [3 Small molecule compounds], [3.7 Methyl (2R)-2-azido-3-hydroxyl-2-methylpropanoate and N-(3-Amino-2-hydroxypropyl)-N-meth-ylsulfuric diamide.], [Paragraph 1].

This sentence previously stated: “[83% of the myxobacteria had antibacterial activity against P. infestans, among which Myxococcus and Coralococcus had a higher antibacterial proportion. The strains with the most significant antibacterial activity of the isolated myxobacteria were M. xanthusB25-I-1, Myxococcus fulvus B25-I-3 and Myxococcus stipitatus X6-II-1. M. xanthus B25-I-1 exhibits antagonistic activity against a variety of fungi and bacteria, and its active extract reduces the content of soluble proteins and the activity of protective enzymes (PPO, POD, PAL and SOD) in P. infestans, Increased oxidative damage and cell membrane permeability. It has a strong inhibitory effect on the hyphae, asexual reproduction and sexual reproduction of P. infestans (Wu, 2018). The active substance of M. fulvus B25-I-3 shows a strong inhibitory effect on the growth of P. infestans, inhibits the growth of mycelium and other asexual reproduction, and reduces the reinfection ability of pathogenic bacteria (Wu, 2018; Wu Z. et al., 2021; Wu Z. H. et al., 2021). After the fermentation products of B25-I-1 and B25-I-3 were separated, it was found that the components that had antagonistic effects on P. infestanscontained methyl (2R)-2-azido-3-hydroxyl-2-methylpropanoate and N- (3-amino-2-hydroxypropyl)-N-meth-ylsulfuricdiamide.]” The corrected sentence appears below: “[Eighty-three percent of the myxobacterial strains were resistant to *P. infestans*, among which the strains of *Myxococcus* and *Coralococcus* accounted for a higher proportion. The strains with the most significant antibacterial activity were *M. xanthus* B25-I-1, *M. fulvus* B25-I-3 and *M. stipitatus* X6-II-1. Strain B25-I-1 exhibited antagonistic activity against a variety of fungi and bacteria, and its active substances reduced the content of soluble proteins and the activity of protective enzymes (PPO, POD, PAL, and SOD) in *P. infestans*, increased oxidative damage and cell membrane permeability. It had a strong inhibitory effect on the hyphae, asexual reproduction and sexual reproduction of *P. infestans* (Wu, 2018). The active substance of strain B25-I-3 showed a strong inhibitory effect on the growth of *P. infestans*, inhibited the growth of mycelium and asexual reproduction, and reduced the infection ability of pathogens (Wu, 2018; Wu Z. et al., 2021; Wu Z. H. et al., 2021). After the fermentation products of B25-I-1 and B25-I-3 were separated, it was found that the components that had antagonistic effects on *P. infestans* contained Methyl(2R)-2-azido-3-hydroxyl-2-methylpropanoate and N- (3-Amino-2-hydroxypropyl)-N-meth-ylsulfuricdiamide.]”

25. A correction has been made to [3 Small molecule compounds], [3.7 Methyl (2R)-2-azido-3-hydroxyl-2-methylpropanoate and N-(3-Amino-2-hydroxypropyl)-N-meth-ylsulfuric diamide.], deleted the second paragraph. The second paragraph has little connect with [3.7 Methyl (2R)-2-azido-3-hydroxyl-2-methylpropanoate and N-(3-Amino-2-hydroxypropyl)-N-meth-ylsulfuric diamide.]
26. A correction has been made to [3 Small molecule compounds], [3.8 Some unknown substances], [Paragraph 1].

The corrected sentence appears below: “[*P. infestans* causes devastating diseases by invading the leaves, stems and tubers of potato plants (Berleman and Kirby, 2009). *M. xanthus* YR-7 isolated from soil samples in Bayannur area of Inner Mongolia had significant resistance to *P. infestans*. The growth inhibition rate of strain YR-7 against *P. infestans* hyphae was as high as 96.67%. The fermentation product of YR-7 was tested in isolated leaves. The experimental results proved that the active substance against *P. infestans* is a non-protein substance (Ren et al., 2016). About 72% of the myxobacteria isolated from soil samples in Ordos and Wuhai areas of Inner Mongolia had varying degrees of antagonistic effects on the growth of *P. infestans*. The ones with stronger ability to inhibit oomycetes were *C. exiguous* E10, *M. fallax* E11 and *C. coralloides* E12. The diameters of the inhibition zones were 26 mm, 24 mm and 24 mm (Ding, 2017; Wu, 2018). About 78.75% of the myxobacteria isolated from soil samples in Alxa area of Inner Mongolia had varying degrees of activity against *P. infestans*. The resistance of *Myxococcus fulvus* AL-24 and *Anqiococcu* AL-10 was outstanding. The fermentation products of strain AL-24 and strain AL-10 had good infection prevention activity and weak infection treatment activity on detached potato leaves (Zhao, 2018). Myxobacteria isolated from Indian soil samples, the extracts of *Coralococcus parvum* S104 and S145 showed broad-spectrum antibacterial activity. The water extract (WE) and DMSO extract (DE) of GNDU172 showed obvious activity against *B. subtilis*, and the DE of strain S213 and strain S223 showed similar activity. The DE of S223 has activity against *Pseudomonas syringae* and *B. cereus* (Kumar et al., 2017). However, all of these active substances are unknow and need to be seperated and identified in the future.]”

27. A correction has been made to [4 Discussion], [Paragraph 1].

[Delete the last sentence of the first paragraph.]

28. A correction has been made to [4 Discussion], [Paragraph 2].

This sentence previously stated: “[One is to degrade the cell wall or increase the permeability of the cell membrane through antibacterial metabolites and cell wall degrading enzymes; the other one is a targeted, contact-dependent killing mechanism through the Tad-like system and protein secretion system.]” The corrected sentence appears below: “[One method is to degrade the cell wall or increase the permeability of the cell membrane through antibacterial metabolites and cell wall degrading enzymes. The other one is a targeted, contact-dependent killing mechanism through the Tad-like system and protein secretion system (Thiery et al., 2022). The Tad-like system mediates the contact-dependent killing of myxobacteria on prey cells (Seef et al., 2021).]”

29. A correction has been made to [4 Discussion], [Paragraph 3].

This sentence previously stated: “[During the interaction between myxobacteria and prey cells, antibiotics, lytic enzymes, hydrolases, etc. may be involved in the process. This review summarizes the prevention and control principles of myxobacteria in plant pathogenic fungi, oomycetes, and bacterial disasters, which can be divided into two categories (Table 1). Myxobacteria secrete carbohydrate-active enzymes that can degrade cell wall components and degrade the components of the cell wall. Myxobacteria produce small molecule compounds that enter the cells of pathogens and inhibit the normal growth and proliferation of pathogens. The CAZymes secreted by myxobacteria are mainly chitinase, β-1,6-glucanase and β-1,3-glucanase, which increase intracellular osmotic pressure by degrading the cell wall of prey cells, promote cell lysis and death. Small molecule compounds produced by myxobacterial fermentation enter prey cells, affecting their normal growth and reproduction or changing their permeability, thereby inducing apoptosis of prey cells.]” The corrected sentence appears below: “[During the interaction between myxobacteria and prey cells, antibiotics, lytic enzymes, hydrolases, etc. involved in the process. This review summarizes the prevention and control principles of myxobacteria in plant pathogenic fungi, oomycetes, and bacterial disasters (Table 1). Myxobacteria secrete enzymes that can degrade cell wall components. Myxobacteria produce small molecule compounds that inhibit the normal growth and proliferation of pathogens. The enzymes secreted by myxobacteria are mainly chitinase, β-1,6-glucanase and β-1,3-glucanase, etc, which increase intracellular osmotic pressure by degrading the cell wall of prey cells, promoting cell lysis and death. Some small molecule compounds produced by myxobacteria affect the normal growth and reproduction of prey cells or changing their permeability, thereby inducing apoptosis of prey cells.]”

30. A correction has been made to [4 Discussion], [Paragraph 4].

This sentence previously stated: “[In the process of preventing and controlling plant diseases with myxobacteria, it is possible to develop a certain biological control agent that inhibits pathogens efficiently, quickly and targetedly, reducing the cost increase and environmental pollution caused by excessive use of pesticides, and maximizing economic and environmental benefits.]” The corrected sentence appears below: “[In the process of preventing and controlling plant diseases, it is possible to use myxobacteria to develop a certain BCAs that inhibits pathogens efficiently, which will be helpful to reduce the cost increase and environmental pollution caused by excessive use of pesticides, and maximize economic and environmental benefits.]”

31. A correction has been made to [4 Discussion], [Paragraph 5].

This sentence previously stated: “[For example, myxobacteria-mediated nanosilver ion paper is used in fruit packaging to extend the shelf life to 15days (Bhople et al., 2016).]” The corrected sentence appears below: “[For example, myxobacteria-mediated paper impregnated with silver nanoparticles (AgNPs) is used in fruit packaging to extend the shelf life to 15 days (Bhople et al., 2016).]”

In the published article, there was an error in the name of Section. Instead of “*[2 Active enzymes]*,” it should be “*[2 Enzymes]*.”

In the published article, there was four errors in the name of Sub-Section. Instead of “*[2.4 Proteases and lipases]*,” it should be “*[2.4 Proteases and peptidases]*.” Instead of “*[3.3 Mucin]*,” it should be “*[3.3 Myxovirescin]*.” Instead of “*[3.4 Cystobactamide analogues and coralmycin A]*,” it should be “*[3.4 Cystobactamid derivatives]*.” Instead of “*[3.6 Protocystin A*,]” it should be “*[3.6 Argyrin derivatives]*.”

In the published article [3.8 Some unknown substances] was not listed in the article.

In the published article, the following 4 citations were deleted in the article, [Müller, S., Strack, S. N., Hoefler, B. C., Straight, P. D., Kearns, D. B., Kirby, J. R., et al. (2014). Bacillaene and sporulation protect Bacillus subtilis from predation by Myxococcus xanthus. Appl. Environ. Microbiol. 80, 5603–5610. 10.1128/aem.01621-14], [Tomura, T., Nagashima, S., Yamazaki, S., Iizuka, T., Fudou, R., and Ojika, M. (2017). An unusual diterpene—enhygromic acid and deoxyenhygrolides from a marine myxobacterium, Enhygromyxa sp. Mar. Drugs 15:109. 10.3390/md15040109], [Velicer, G. J., and Vos, M. (2009). Sociobiology of the myxobacteria. Annu. Rev. Microbiol. 63, 599–623. 10.1146/annurev.micro.091208.073158], [Zeigler, D. R., Pragai, Z. N., Rodriguez, S., Chevreux, B., Muffler, A., Albert, T., et al. (2008). The origins of 168, W23, and other Bacillus subtilis legacy strains. J. Bacteriol.190, 6983–6995. 10.1128/jb.00722-08.]

The authors apologize for these errors and state that this does not change the scientific conclusions of the article in any way. The original article has been updated.
