# Supplementary figures and images for: Active substances of myxobacteria against plant diseases and their action mechanisms
Source: Front Microbiol. 2024 Jan 8;14:1294854. doi: 10.3389/fmicb.2023.1294854 (PMC10800785; doi:10.3389/fmicb.2023.1294854)

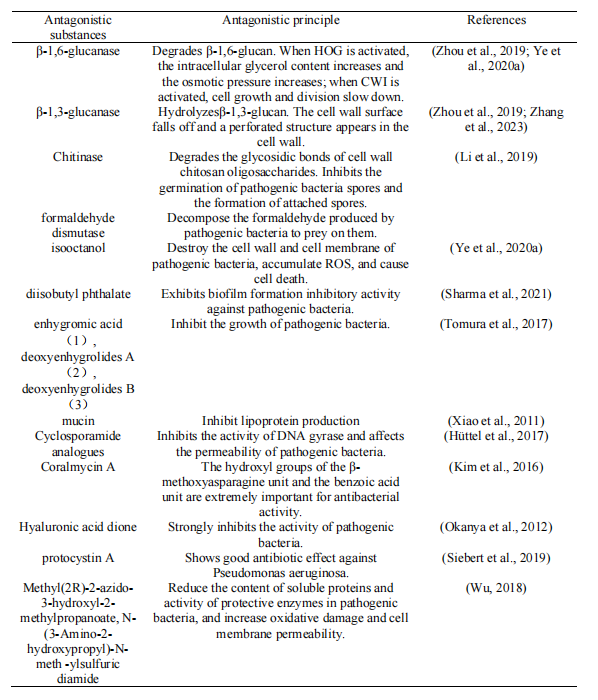

Supplement: Supplementary file 1 [file Image_1.PNG]
